# Widespread Rotavirus H in Commercially Raised Pigs, United States

**DOI:** 10.3201/eid2007.140034

**Published:** 2014-07

**Authors:** Douglas Marthaler, Kurt Rossow, Marie Culhane, Sagar Goyal, Jim Collins, Jelle Matthijnssens, Martha Nelson, Max Ciarlet

**Affiliations:** University of Minnesota Veterinary Diagnostic Laboratory, Saint Paul, Minnesota, USA (D. Marthaler, K. Rossow, M. Culhane, S. Goyal, J. Collins);; University of Leuven, Leuven, Belgium (J. Matthijnssens);; Fogarty International Center of the National Institutes of Health, Bethesda, Maryland, USA (M. Nelson);; Novartis Vaccines and Diagnostics, Inc., Cambridge, Massachusetts, USA (Max Ciarlet)

**Keywords:** RVH, novel rotavirus, phylogenetic analysis, pigs, swine, viruses, United States

## Abstract

We investigated the presence in US pigs of rotavirus H (RVH), identified in pigs in Japan and Brazil. From 204 samples collected during 2006–2009, we identified RVH in 15% of fecal samples from 10 US states, suggesting that RVH has circulated in the United States since 2002, but probably longer.

Rotaviruses (RVs) belong to the *Reoviridae* family and are a major cause of severe diarrhea in humans and animals worldwide (*1*). According to the International Committee on Taxonomy of Viruses, the *Rotavirus* genus is divided into 5 antigenically distinct groups or species (RVA, RVB, RVC, RVD, RVE), 2 tentative species (RVF, RVG), and an unassigned species (ADRV-N), recently confirmed to be distinct from the other RV species, and now referred to as RVH (*2*,*3*).

Three human RVH strains from Asia (ADRV-N, J19, B219) (*4*–*8*) and a porcine RVH strain (SKA-1) (*9*) were identified during 1997–2002. In 2012, three Brazil porcine RVH strains BR63, BR60, and BR59 (GenBank accession nos. KF021621, KF021620, and KF021619) were identified, bringing to only 7 the total number of known RVH strains. To investigate the presence of RVH in US swine, we screened 204 porcine samples collected during 2006–2009.

## The Study

We identified RVH in a porcine intestinal sample (RVH/Pig-wt/USA/AR7.10-1/2012/GXP[X]) submitted from a farm in Arkansas in 2012. Subsequently, we rescreened 204 available RVA-, RVB-, and/or RVC-positive porcine samples collected during 2006–2009 from 16 US states for RVH. The samples were from 5 different age groups of pigs: 1–3 days (21 samples), 4–7 days (23), 8–20 days (19), 21–55 days (110), and >55 days (9); 22 samples were from pigs of unknown age. Sample selection, histologic examination, extraction of genomic material, reverse transcription PCR (RT-PCR) amplification, sequencing of viral protein (VP) 6 gene, and statistical and sequence analysis are described in the online Technical Appendix (http://wwwnc.cdc.gov/EID/article/20/7/14-0034-Techapp1.pdf).

**Figure 1:**
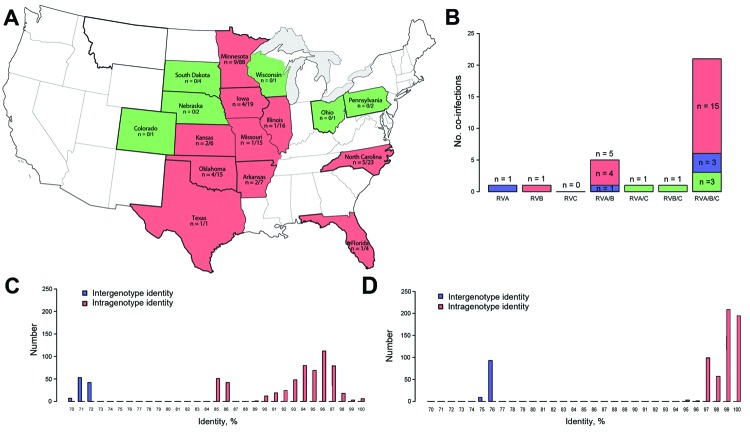
Epidemiologic and molecular distribution of porcine rotavirus H (RVH) strains, United States, 2006–2009. A) Geographic distribution of RVH-positive porcine samples/total number of samples tested. Pink indicates states containing positive samples; green indicates states negative samples; white indicates states from which samples were not submitted. B) Distribution of RVH-positive samples and age group in pigs co-infected with RVA, RVB, and/or RVC. Blue indicates samples from the 4–20-day age group; pink indicates samples from the 21–55-day age group; green indicates samples from the >55-day age group. C) RVH viral protein 6 nt pairwise identity. D) RVH amino acid pairwise identity.

We identified RVH in 30 (15%) of the 204 samples, including sample AR7.10-1 (online Technical Appendix Table). RVH strains were identified in samples from 10 US states (Figure 1, panel A). The first US sample was identified on November 7, 2006. Of samples from age groups in which we detected positive results, most (20/111, 18%) were from 21–55-day-old pigs; RVH was not detected in 1–3-day-old piglets. We also detected RVH-positive samples in 4–20-day-old (5/42, 12%) and >55-day-old (5/9, 56%) pigs. The number of positive and negative samples differed significantly between age groups (p = 0.036, Fisher exact test). The odds of 21–55 day-old pigs being RVH positive was not significant (odds ratio [OR] 1.63, p = 0.36); however, in the >55-day group, the odds of being RVH positive was significant (OR 5.92, p = 0.031), compared with odds for the 4–20-day group. The trend for increased RVH positivity by age group was not significant (p = 0.94, Wald χ^2^ test).

Although we identified only 5 samples with RVH in pigs co-infected with RVA and RVB, co-infections with RVH and RVA, RVB, both RVA and RVC, or both RVB and RVC (1 sample each) also were identified but did not differ significantly (p>0.05, Fisher exact test) (Figure 1, panel B). We did not identify RVH co-infected with only RVC. Most RVH samples (21 [70%]) were identified from pigs co-infected with RVA, RVB, and RVC, which was significantly higher from any other RVH co-infections with RVA, RVB, RVC, RVAB, RVAC, or RVBC (p<0.001, Fisher exact test). Of these 21 RVA, RVB, RVC, and RVH co-infected samples, 15 were from 21–55-day-old pigs (Figure 1, panel B).

**Figure 2:**
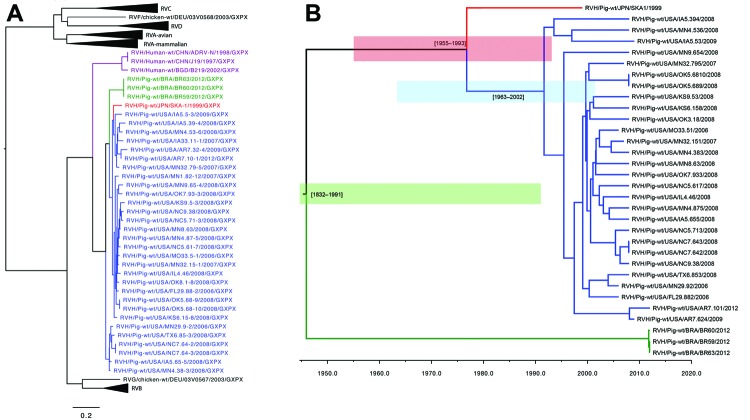
A) Nucleotide neighbor-joining phylogenetic tree of rotavirus (RV) A–D and F–H viral protein (VP) 6 sequences. Blue strains are from the United States; green strains are from Brazil; and the red strain is from Japan. Purple strains are from humans. Scale bar indicates percentage of dissimilarity between sequences. B) Time-scaled phylogeny of swine RVH VP6 sequences using a Bayesian Markov chain Monte Carlo approach. Blue shaded region indicates the time from the most recent common ancestor range (tMRCA) of the US strain; red shaded region indicates the US and Japan RVH tMCRA range; green shaded region indicates the tMRCA range for all swine RVH VP6 sequences.

The US porcine RVH VP6 sequences (GenBank accession nos. KF757260–KF757289) exhibited 91%–100% nt identity with each other and shared 89%–92% nt identity with Japan porcine strain SKA-1 and 85%–87% nt identity with Brazil porcine strains BR63, BR60, and BR59 (Table 1). The US porcine and human RVH VP6 sequences shared 70%–73% nt identity. The US porcine RVH VP6 sequences were 97%–100% aa identical with each other and 97%–98% and 96%–98% aa identical with the Japan and the Brazil porcine strains, respectively. The US porcine and human RVH VP6 sequences were 75.3%–76.8% aa identical (Table 1). The nucleotide and amino acid pairwise identity charts (Figure 1, panels C and D) and phylogenetic trees (Figure 2, panel A) suggest the existence of at least 2 distinct RVH VP6 (I) clusters/genotypes containing human and porcine strains, respectively.

**Table 1:** Nucleotide and amino acid percentage identities of RVH*

RVH type	US porcine RVH, %	Japan porcine RVH, %	Brazil porcine RVH, %	Human RVH, %
US porcine RVH				
Nucleotide	91–100	89.2–91.9	85.2–86.8	70.4–72.8
Amino acid	97–100	96.5–98.2	95.7–97.7	75.3–76.8
Japan porcine RVH				
Nucleotide	89.2–91.9	NA	85.5	71.7–72.3
Amino acid	96.5–98.2	NA	97	76.5-76.8
Brazil porcine RVH				
Nucleotide	85.2–86.8	85.5	100	71.1–71.2
Amino acid	95.7–97.7	97	100	75.8-76
Human RVH				
Nucleotide	70.4–72.8	71.7-72.3	71.1-71.2	94–100
Amino acid	75.3–76.8	76.5–76.8	75.8–76	98.7–100

*RVH, rotavirus H; NA, not applicable

Compared with other RV species, the US RVH VP6 sequences shared the highest nucleotide and amino acid identities with RVG (51%–53% and 39%–41%, respectively) and RVB (47%–52% and 34%–39%, respectively) (Table 2). In the RV VP6 phylogenetic tree, The RVH, RVG, and RVB VP6 sequences clustered in 1 large branch, whereas the RVA, RVC, RVF, and RVD sequences clustered separately in another large branch (Figure 2, panel A). The RVH evolutionary rate (substitution/site/year) from BEAST (http://tree.bio.ed.ac.uk/) was estimated at 2.6 × 10^−3^ (95% CI 5.83 × 10^−4^ to 4.46 × 10^−3^). On the basis of the estimate of the time from the most recent common ancestor for the VP6 gene segment, we believe that US RVH strains circulated in US swine for at least a decade and possibly much longer (the time from the most recent common ancestor 1963–2002, 95% highest posterior density [HPD]) (Figure 2, panel B). The US and Japan RVH VP6 sequences diverged during 1955–1993, 95% HPD, and the estimated divergence of the Brazil RVH VP6 sequences from the US and Japan RVH VP6 sequences was 1832–1991, 95% HPD.

**Table 2:** Nucleotide and amino acid percentage identities of RVs*

RV type	RVA	RVB	RVC	RVD	RVF	RVG	RVH
RVA							
Nucleotide	65.2–100	29.7–36.2	48.5–55.7	46.4–52.1	46.3–50.8	32.9–36.7	31.7–36.2
Amino acid	65–100	7.5–11.3	36.3–42.9	33.3–39.9	31.8–37.2	11.1–13.5	9.9–13.1
RVB							
Nucleotide	29.7–36.2	64.8–100	30.5–34.4	29.2–32.9	30.1–32.9	50.7–57.1	47.4–51.7
Amino acid	7.5–11.3	66.2–100	10.6–13.9	10.4–12.7	11.3–13.4	46.1–49.4	34.4–39.4
RVC							
Nucleotide	48.5–55.7	30.5–34.4	81.4–100	47.2–49.8	47.4–48.3	33.8–34.2	31.5–34.6
Amino acid	36.3–42.9	10.6–13.9	87.1–100	34.7–35.4	32.7–33.9	14.4–14.6	13.4–14.7
RVD							
Nucleotide	46.4–52.1	29.2–32.9	47.2–49.8	90.1–99.6	49.8–50.7	33–34	31.9–34.4
Amino acid	33.3–39.9	10.4–12.7	34.7–35.4	98.2–99.7	36.6–37.6	12–12.5	14.5–16.8
RVF							
Nucleotide	46.3–50.8	30.1–32.9	47.4–48.3	49.8–50.7	NA	32.3	31–32.2
Amino acid	31.8–37.2	11.3–13.4	32.7–33.9	36.6–37.6	NA	11.1	12.6–14
RVG							
Nucleotide	32.9–36.7	50.7–57.1	33.8–34.2	33–34	32.3	NA	50.7–52.2
Amino acid	11.1–13.5	46.1–49.4	14.4–14.6	12–12.5	11.1	NA	39.1–41.4
RVH							
Nucleotide	31.7–36.2	47.4–51.7	31.5–34.6	31.9–34.4	31–32.2	50.7–52.2	70.4–100
Amino acid	9.9–13.1	34.4–39.4	13.4–14.7	14.5–16.8	12.6–14	39.1–41.4	75.3–100

*RV, rotavirus; NA, not applicable.

## Conclusions

Our data indicate that RVH is widespread in US swine herds. Although the samples analyzed already were known to be positive for RV species A, B, and/or C, our identification of RVH in 15% of samples is remarkable. In the United States, piglets are weaned at 21 days of age and then mixed with other piglets from different production sites, which may explain the higher rate of RV co-infections in 21–55-day-old pigs (*10*,*11*). These findings suggest that RVH is underdiagnosed in US swine herds and requires further surveillance.

Our phylogenetic analysis indicates that the RVH strains circulating in US swine is evolutionarily distinct from that found in humans, as well as from swine in Brazil and Japan. Although our low sample number and sequencing of a single gene (VP6) makes the genetic diversity of RVH in US swine herds difficult to fully assess, the lack of spatial structure in the tree indicates extensive gene flow of RVH between swine herds in different US regions. Inferring the circulation of RVH in US swine herds is difficult because of the small sample size, although our time-structured phylogenetic analysis indicates at least 1 decade of circulation. Although US swine are routinely transported to South America, the phylogeny indicates that the VP6 gene of US swine RVH viruses is more closely related to that of Japan strain SKA-1 than to those of the 3 Brazil strains included in this analysis.

In conclusion, we identified RVH in 30 samples from pigs co-infected with RVA, RVB, and/or RVC in the United States, which indicates that RVH has been circulating in US swine for at least 1 decade and perhaps for longer. The human and porcine RVH VP6 sequences clustered into separate branches in the phylogenetic tree, but the presence of RVH in swine clearly raises the possibility of interspecies transmission. Because the swine samples were co-infected with RVA, RVB, and/or RVC, the role of RVH in pathogenesis remains unknown but this circumstance illustrates the need for molecular epidemiologic studies.

Technical Appendix Sample selection, histologic examination, extraction of genomic material, reverse transcription PCR amplification, sequencing of viral protein 6 gene, and statistical and sequence analysis of rotavirus H, United States, 2006–2009.
