# Supplementary material for: Widespread Rotavirus H in Commercially Raised Pigs, United States
Source: Emerg Infect Dis. 2014 Jul;20(7):1203–6. doi: 10.3201/eid2007.140034 (PMC4073875; doi:10.3201/eid2007.140034)
Supplement: Technical Appendix — Sample selection, histologic examination, extraction of genomic material, reverse transcription PCR amplification, sequencing of viral protein 6 gene, and statistical and sequence analysis of rotavirus H, United States, 2006–2009. [file 14-0034-Techapp-s1.pdf]

Article DOI: http://dx.doi.org/10.3201/eid2007.140034

Widespread Rotavirus H in Commercially Raised Pigs, United States

Technical Appendix

Sample Selection

The University of Minnesota Veterinary Diagnostic Laboratory routinely receives porcine intestinal or fecal samples from pig farms across the United States to determine the causative agents of gastrointestinal disease, which are screened by reverse transcription PCR (RT-PCR) for transmissible gastroenteritis coronavirus; rotavirus (RV) A, RVB, and RVC; and a variety of bacterial pathogens by bacterial cultures (*1*,*2*). In June 2012, a porcine intestinal sample (MN123) from a pig on a farm in Minnesota showed histologic RV-like lesions (blunt or nude villus tips) by light microscopy, but the sample was negative for RVA, RVB, and RVC by RT-PCR. Another porcine intestinal sample (AR7.10-1) from a pig on a farm in Arkansas was positive for RVA, RVB, and RVC. Both samples (MN123 and AR7.10-1) were screened for RVH with RVH viral protein (VP) 7–, VP6-, and nonstructural protein (NSP) 4-specific primers. The porcine intestinal sample MN123 was negative for RVH with all primer sets; however, the AR7.10-1 sample was positive for RVH with the RVH VP6-specific primers. The RVH VP6-specific primers amplified a band of ≈1,200 nt, and sequencing of the band revealed a 90% nt identity to the swine Japanese RVH SKA-1 strain, identifying an RVH strain for the first time in the United States. From a previous RV study, the extracted RNA from 204 RVA-, RVB- and/or RVC-positive samples collected during 2006–2009 was readily available and used to determine whether RVH also was present in those samples. For the screening of RVH, only the RVH VP6-specific primers were used because the RVH VP7- and NSP4-specific primers had failed to detect RVH in the sample AR7.10-1.

Histology, Extraction of Genomic Material, and RT-PCR Amplification

Samples of small intestine were fixed in 10% neutral buffered formalin, embedded in paraffin, and stained by using Harris' hematoxylin and eosin, as described previously (*1*,*2*). Porcine intestinal or fecal samples were homogenized with Hyclone donor equine serum (Thermo Fisher Scientific, Waltham, MA, USA) at a 2:3 ratio for 15 min, then underwent 3,000 × g centrifugation for 1 h, as described previously (*1*,*2*). The total nucleic acid was extracted by using the Ambion MagMax 1836 extraction kit following the manufacturer's instructions (Life Technologies, Carlsbad, CA, USA).

For the molecular detection of RVH strains, VP7, VP6, and NSP4-specific primers were designed (forward RVH_VP7_1-20: 5′-GGCAATTTGAAGCCATGTTG-3′ and reverse RVH_VP7_804-781: 5′-CATAACGGATTTCTCAACGTTATG-3′, forward RVH_VP6_1-23: 5′-GGCAATTTCTTGCTACAAGTGAC-3′ and reverse RVH_VP6_1203-1181: 5′-GGGTATATTTTATTTGCTATACTACTACGG-3′, and forward RVH_NSP4_1-24: 5′-GGCATTTTGTTCATCACAAATCACG-3′ and reverse RVH_NSP4_721-698: 5′-CTACCAAGCTATGTTTCCATCCAT-3′) based on sequence alignment with ClustalW of the VP7, VP6, and NSP4 sequences of the single porcine (SKA-1) and the 3 human (ADRV-N, J19, B219) RVH strains (*3*–*8*). The RT-PCR reaction used the QIAGEN OneStep RT-PCR kit (QIAGEN. Valencia, CA, USA) in accordance with the manufacturer's recommended instructions and following the previously described thermal cycling conditions (*1*,*2*). RT-PCR products were visualized, purified, and Sanger sequenced by using previously described methods (*1*,*2*). The nucleotide peaks from the traces files were visually evaluated and had a minimum of 2 times consensus coverage in Lasergene Seqman 10.0 program (DNASTAR, Madison, WI, USA).

In Geneious Pro (http://www.geneious.com)Tre, the novel, and GenBank RVH (SKA-1, AB576626; B219, DQ168033; ADRV-N, AY632080; J19, DQ113902; BR63, KF021621; BR60, KF021620; BR59, KF021619) RV VP6 nucleotide sequences were aligned with ClustalW (*8*). The nucleotide and amino acid phylogenetic trees were constructed with the neighbor-joining method (*9*). A time-scaled phylogeny was inferred for the swine RVH VP6 sequences by using a Bayesian Markov Chain Monte Carlo (MCMC) approach, available in the BEAST package (BEASTv1.8.0) (*10*–*16*). A general-time reversible model of nucleotide substitution was implemented, with a γ distributed among-site rate variation. Using a relaxed molecular clock and

a Bayesian skyline population prior, the MCMC chain was run for 300 million generations, with subsampling every 30,000 iterations. The initial 10% of the chain was discarded as burn-in, and a maximum clade credibility tree was summarized by using TreeAnnotator (v.1.8.0) (*10–16*). Times to the most recent common ancestor were identified on key nodes using FigTree (v1.4.0) (http://beast.bio.ed.ac.uk/software/figtree/).

Statistical Analysis

The Fisher exact test was used to determine whether the number of positive and negative samples significantly differed among age groups. By using the age groups that contained RVH-positive samples (4–20-day, 21–55-day, and >55-day age groups), a logistic regression model was used to estimate the odds of RVH infection by age group, and the trend of RVH infection by age was tested by using Wald χ^2.

Sequence Analysis

The open reading frame alignment (1,191 nt) of the 3 human (ADRV-N, J19, B219), 4 porcine (SKA-1, BR63, BR60, BR59), and the 30 novel porcine US RVH VP6 sequences revealed 682 (37%) identical sites. When compared with the human RVH sequences, the US porcine RVH VP6 sequences had 2 nt deletions at 1223 and 1224 in the 3′ untranslated region. The RVH VP6 396 amino acid alignment of the US (n = 30), Chinese (n = 2), Bangladeshi (n = 1), Japanese (n = 1), and Brazilian (n = 3) strains revealed 291 (74%) identical sites. Although most of the identified polymorphisms involved 2 different amino acids, polymorphisms involving 3 amino acids were identified at positions 44, 48, 150, 201, 220, 258, 353, and 378 (data not shown). Sequencing of the 30 novel RVH strains revealed 6 samples (IA5.5-3, MN4.53-6, MN4.87-5, MN32.15-1, AR7.32-4, and AR7.10-1) that contained mixed RVH infections or viral population, as indicated by ambiguous nucleotides within each the sequences (data not shown). Each ambiguous nucleotide translated only 1 aa.

Nucleotide Sequence Accession Numbers

The 30 RVH VP6 sequences were deposited into GenBank under accession nos. KF757260–KF757289.

Rotavirus VP6 Nucleotide Sequences Used in the Phylogenetic Analysis

CMH8/01, EU372728; CMH38/02, EU372740; CMH52/01, EU372731; CMH127/01, EU372733; CMH66/02, EU372743; CMH187/01, EU372736; CMH101/01, EU372732; CMH82/02, EU372745; CMH71/02, EU372744; CMH95/02, EU372747; CMH85/02, EU372746; CMH017/05, GU288638; CMH9/02, EU372739; CMH49/02, EU372741; CMH4/02, EU372738; RVA/Human-wt/BEL/B3458/2003/G9P[8], DQ870504; CMH151/01, EU372735; CMH202/01, EU372737; CMH55/02, EU372742; CMH77/00, EU372727; CMH186/01, EU372750; CMH37/01, EU372730; CMH16/01, EU372729; CMH142/01, EU372734; CMH5/00, EU372724; CAU05202, JF766582; CAU202, EU556223; RVA/Human-wt/AUS/CK00100/2010/G1P[8], JX027973; RVA/Human-wt/AUS/CK00096/2010/G1P[8], JX027939; RVA/Human-wt/AUS/CK00099/2010/G1P[8], JX027961; RVA/Human-wt/AUS/CK00097/2010/G1P[8], JX027952; RVA/Human-wt/Bel/BE00098/2009/G1P[8], JN258930; OH2024, AB669018; OH1998, AB669014; CAU09371, JF766593; RVA/Human-wt/AUS/CK00066/2007/G1P[8], KC769386; human/Victoria/CK00029/2006/G1P[8], JF490364; OH1889, AB669006; OH1908, AB669010; ISO13, EF472944; RVA/Human/NCA/7J/2010/G1P[8], JN129098; RVA/Human/NCA/9J/2010/G1P[8], JN129099; RVA/Human-wt/AUS/CK00088/2009/G1P[8], JX027875; RVA/Human-wt/BEL/BE00035/2008/G1P[8], HQ392305; ISO34, EF472946; ISO94, EF472948; ISO912, EF472951; RVA/Human-wt/USA2009727051/2009/G9P[8], HM773628; RVA/Human-wt/USA2009727047/2009/G9P[8], HM773617; RVA/Human-wt/USA/2009727093/2009/G9P[8], HM534677; RVA/Human-wt/USA2009727036/2009/G9P[8], HM773595; human/Bethesda/DC1/2009/G9P[8], HQ702212; human/Bethesda/DC8/2009/G9P[8], HQ702256; RVA/Human-wt/USA2007719825/2007/G1P[8], HM773749; mcs/1007, EU753972; RVA/Human-wt/ZAF/MRCDPRU2061/2010/G1P[8], KF636183; RVA/Human-wt/ZAF/MRCDPRU1492/2010/G1P[8], KF636194; RVA/Human-wt/ZAF/MRCDPRU2030/2010/G1P[8], KF636205; RVA/Human-wt/ZAF/MRCDPRU1544/2010/G1P[8], KF636216; RVA/Human-wt/ZAF/MRCDPRU2052/2010/G1P[8], KF636282; RVA/Human-wt/ZAF/MRCDPRU2035/2010/G1P[8], KF636238; RVA/Human-wt/BGD/Dhaka6/2001/G11P[25], GU199521; RVA/Human-wt/BGD/Dhaka6/2001/G11P[25], EF560707; RVA/Human-wt/USA/2007719635/2007/G1P[8], JN258370; CU537KK/09, JN706553; RVA/Human/NCA/64J/2010/G3P[8], JN129108; RVA/Human/NCA/125L/2010/G3P[8], JN129110; human/Vanderbilt/VU080922/2008/G3P[8], JF491057; RVA/Human-wt/USA2008747322/2008/G3P[8], HM773738; BJCR4916, GU947708; BJCR5317, GU947705; CU328NR/08, JN706540; CU329NR/08, JN706541; RVA/Human-wt/BGD/Matlab36/2002/G11P[8], GU199507; US9828, EF426139; RVA/Human-wt/ZWE/MRCDPRU1708/2009/G9P[8], KF636304; MRCDPRU4677, JN605430; MRCDPRU1723, JN605419; RVA/Human-wt/ZAF/2371WC/2008/G9P[8], JN014004; RVA/Human-wt/ZAF/2371WC/2008/G9P[8], JN014005; Z1108, JF813105; LB2719, HM467946; RVA/Human-wt/AUS/CK00081/2007/G1P[8], KC195773; US6153, EF426121; human/Victoria/CK00014/2004/G1P[8], JF490210; human/Victoria/CK00011/2004/G1P[8], JF490188; human/Victoria/CK00008/2004/G1P[8], JF490166; RVA/Human-wt/AUS/CK00074/2007/G1P[8], JX027753; RVA/human-wt/JPN/OH3592/2012/G1P[8], AB796453; human/Victoria/CK00037/2006/G1P[8], JF490440; human/Vanderbilt/VU060710/2006/G1P[8], JF490903;

human/Vanderbilt/VU06071/2006/G1P[8], JF490881; RVA/Human-wt/USA2007744270/2007/G1P[8], HM773826; US6668, FJ152131; LB2771, HM467950; human/Vanderbilt/VU050616/2005/G1P[8], JF490605; human/Vanderbilt/VU050647/2005/G1P[8], JF490739; US9951, EF426129; US9825, EF426128; US6097, EF426134; RVA/Human-wt/Bel/BE00068/2000/G1P[8], JN258814; US8673, EF426135; US8675, EF426137; US8674, EF426136; US8979, EF426126; US6161, EF426122; US8970, EF426125; US8616, EF426138; RVA/Human-wt/AUS/CK00094/2009/G1P[8], JX027918; RVA/Human-wt/AUS/CK00092/2009/G1P[8], JX027907; RVA/Human-wt/AUS/CK00091/2009/G1P[8], JX027897; human/Vanderbilt/VU050672/2005/G12P[8], JF790293; human/Vanderbilt/VU050674/2005/G12P[8], JF790304; US6597, FJ152120; RVA/Human-wt/BEL/BE00022/2007/G1P[8], HQ392171; RVA/Human-wt/BEL/BE00019/2006/G1P[8], HQ392139; RVA/Human-wt/BEL/BE00007/2005/G1P[8], HQ392029; RVA/Human-wt/BEL/BE00006/2005/G1P[8], HQ392017; RVA/Human-wt/BEL/BE00015/2006/G1P[8], HQ392094; RVA/Human-wt/BEL/BE00046/2008/G1P[8], HQ392438; RVA/Human-wt/AUS/CK00061/2007/G1P[8], KC769353; RVA/Human-wt/Bel/BE00076/2001/G1P[8], JN258839; RVA/Human-wt/ZAF/MRCDPRU1262/2004/G1P[8], KF636227; RMC/G66, AY601553; RMC437, AY601554; RVA/Human-wt/AUS/CK00095/2010/G1P[8], JX027929; RVA/Human-wt/JPN/OH3514/2012/G1P[8], AB796452; CAU09376, JF766604; RUSNov05640, JQ248937; Nov05K25, GU138212; RVA/Human-wt/ZAF/3133WC/2009/G12P[4], HQ657153; RVA/Human-wt/ZAF/3176WC/2009/G12P[6], HQ657164; Omsk08478, GQ477115; E1545, JF813101; RVA/Human-tc/CHN/Y128/2004/G1P[8], JQ087427; RVA/Human-tc/E1911/2009/G1P[8], JQ087449; CU769KK/10, JN706549; Nov09D67, GU592515; Hu/RUS/Nov09D89/G4P[8], JQ230069; CMH150/01, EU372748; human/Vanderbilt/VU08097/2008/G3P[8], JF490992; RVA/Human-wt/USA2009726997/2009/G3P[8], HM773727; RVA/Human-wt/USA2008747369/2008/G3P[8], HM773661; Nov09D249, GU390459; Hu/RUS/Nov09D296/P[8], JQ230079; Nov09B28, GQ477117; Hu/RUS/Nov09D381/2009/G1P[8], JQ585601; Hu/RUS/Nov09D386/G1P[8], JQ230083; Nov10N186, HQ611004; Nov10N71, HQ611001; Hu/RUS/Nov09D278/G1P[8], JQ230068; Hu/RUS/Nov10N218/G1P[8], JQ230086; Nov09D58, GQ477129; RVA/Human-wt/USA2008747112/2008/G3P[8], HM773716; Nov09D27, GQ477126; Veitnam/163/G10P[8], AB714263; RVA/Human-wt/AUS/CK00062/2007/G1P[8], JX027706; CU460KK/09, JN706542; RVA/Human-wt/AUS/CK00087/2009/G1P[8], JX027862; Hu/RUS/Nov09D189/G1P[8], JQ230073; Hu/RUS/Nov09D187/G1P[8], JQ230072; Hu/RUS/Nov10N735/2010/G1P[8], JQ585604; RVA/Humantc/CHN/R588/2005/G1P[8], JQ087438; human/Victoria/CK00047/2006/G1P[8], JF490519; RVA/Human-wt/AUS/CK00059/2007/G1P[8], KC769342; RVA/Human-wt/AUS/CK00056/2007/G1P[8], JX027685; CAU164, EU679386; Hu/NhaTrang/V17/2006/VNM, AB605598; CMH042/07, JQ043295; RUSNov083123, GQ477096; Nov083281, GQ477099; human/Vanderbilt/VU080924/2008/G3P[8], JF491068; Nov083404, GQ477102; CMH014/07, JQ043292; RVA/Human-wt/USA/2007719674/2007/G1P[8], JN258359; Omsk07217, GQ477109; Nov09D23, GQ477125; Nov09D110, GU390454; human/Vanderbilt/VU08099/2008/G3P[8], JF491003; human/Vanderbilt/VU080912/2008/G3P[8], JF491014; human/Vanderbilt/VU080927/2008/G3P[8], JF790334;

human/Vanderbilt/VU08095/2008/G3P[8], JF490970; human/Vanderbilt/VU080925/2008/G3P[8], JF491079; RVA/Human-wt/USA2008747337/2008/G3P[8], HM773672; RVA/Human-wt/USA2008747336/2008/G3P[8], HM773683; human/Vanderbilt/VU080930/2008/G3P[8], JF491124; RVA/Human-wt/USA2008747500/2008/G3P[8], HM773650; CMH185/01, EU372749; US0468, EF426120; human/Vanderbilt/VU060721/2006/G3P[8], JF490915; CU976KK/11, JN706547; CU938BK/11, JN706548; CU747KK/10, JN706546; CU766KK/10, JN706545; Nov10N243, HQ611007; Nov10N660, HQ611027; CMH032/05, GU288639; CMH015/05, GU288637; RVA/Human-wt/BGD/Dhaka16/2003/G1P[8], DQ492673; RVA/Human-wt/BGD/Dhaka25/2002/G12P[8], DQ146653; CU616TK/09, JN706544; CU615TK/09, JN706543; 6361, HQ609561; ISO99, EF472950; RVA/Human-wt/AUS/CK20043/2010/G1P[8], KC443492; RVA/Human-wt/BEL/BE00039/2008/G1P[8], HQ392348; RVA/Human-wt/USA2007719739/2007/G1P[8], HM773760; RVA/Human-wt/ITA/JES11/2010/G9P8, JX195089; RVA/Human-wt/BEL/BE00017/2006/G1P[8], HQ392118; RVA/Human-wt/BEL/BE00034/2008/G1P[8], HQ392296; RVA/Human-wt/BEL/BE00040/2008/G1P[8], HQ392361; RVA/Human-wt/ZAF/MRCDPRU2330/2009/G1P[8], KF636271; RVA/Human-wt/ZAF/MRCDPRU2306/2009/G1P[8], KF636172; RVA/Human-wt/ZAF/MRCDPRU2325/2009/G1P[8], KF636161; RVA/Human-wt/BEL/BE00043/2009/G1P[8], HQ392383; 61060, HQ609563; RVA/Human-wt/AUS/CK00083/2008/G1P[8], JX027820; ISO25, EF472945; Omsk08416, HQ738593; Hu/RUS/Nov09D210/G1P[8], JQ230071; human/Vanderbilt/VU08096/2008/G12P[8], JF490981; human/Vanderbilt/VU080939/2008/G12P[8], JF790348; RVA/Human-wt/Bel/BE00097/2009/G1P[8], JN258919; Hu/MAL23/G1P[8], JN591409; Hu/MAL12/G12P[6], JN591408; ISO92, EF472947; Hu/RUS/Nov10N921/P[6], JQ230095; GER17208, FJ747629; RVA/Human-wt/AUS/CK00089/2009/G1P[8], JX027886; CU331NR/08, JN706532; RVA/Human/BGD/Matlab13/2003/G12P[6], DQ146675; RVA/Human-wt/BGD/Dhaka12/2003/G12P[6], DQ146664; human/Victoria/CK00006/2004/G1P[8], JF490145; RVA/Human-wt/Bel/BE00092/2003/G1P[8], KC193637; Hu/RUS/Nov10N611/G1P[8], JQ230094; Nov09D74, GQ477130; Hu/NhaTrang/V32/2006/VNM, AB605601; Hu/NhaTrang/V30/2006/VNM, AB605600; Hu/NhaTrang/V20/2006/VNM, AB605599; RVA/Human-wt/BEL/BE00038/2008/G1P[8], HQ392338; RVA/Human-wt/Bel/BE00073/2001/G1P[8], JN258829; RMC100, AF531912; RMC83, AY601550; RMC61, AY601549; RVA/Human-wt/CMR/MRCDPRU1417/2009/G9P[8], KF636293; Hu/MAL82/G9P[8], JN591411; MRCDPRU2427, JN605452; human/Vanderbilt/VU06077/2006/G1P[8], JF490892; human/Vanderbilt/VU060735/2006/G1P[8], JF490956; human/Vanderbilt/VU060727/2006/G1P[8], JF490925; human/Vanderbilt/VU050669/2005/G1P[8], JF490805; RVA/Human-wt/BEL/BE00023/2007/G1P[8], HQ392184; RVA/Human-wt/BEL/BE00036/2008/G1P[8], HQ392316; RVA/Human-wt/BEL/BE00029/2008/G1P[8], HQ392237; MMC38, EU979380; MMC71, EU979383; SK277, EU839971; SK423, EU839965; RVA/Human-wt/AUS/CK00064/2007/G1P[8], KC769364; human/Victoria/CK00001/2004/G1P[8], JF490090; human/Victoria/CK00002/2004/G1P[8], JF490100; human/Victoria/CK00012/2004/G1P[8], JF490200; Nov04H308, GU390417;

Hu/RUS/Nov04H318/2004/G1P[8], JQ818164; Hu/RUS/Nov03H251/2003/G1P[8], JQ818160; Nov03H7, GU390414; RVA/Human-wt/Bel/BE00079/2002/G1P[8], JN258847; Nov072799, HQ445976; RVA/Human-wt/BEL/BE00003/2004/G9P[8], HQ391996; RVA/Human-wt/AUS/CK20019/2001/G1P[8], KC443580; Nov0582, GU390429; RVA/Human-wt/Croatia/CR2006/2006/G8P[8], JQ988903; Nov10N205, HQ611006; Nov10N4, HQ610998; Omsk0778, GU138216; Hu/RUS/Nov10N351/G4P[8], JQ230088; Hu/RUS/Nov09D354/G4P[8], JQ230082; Nov071805, GQ477089; Nov10N429, HQ611015; Hu/RUS/Nov10N405/G4P[8], JQ230089; Omsk08459, HQ738586; Omsk08421, HQ738595; Omsk08362, GU390447; Hu/RUS/Omsk08254/2008/G4P[8], JQ818151; Omsk08377, GU390448; Omsk08246, HQ738584; Nov09D12, GQ477124; MRCDPRU1424, JN605408; RVA/Human-wt/ITA/ASTI23/2007/G9P8, JX185762; RVA/Human-wt/BEL/BE00049/2009/G1P[8], HQ392457; RVA/Human-wt/BEL/BE00037/2008/G1P[8], HQ392327; RVA/Human-wt/BEL/BE00047/2009/G1P[8], HQ392448; RVA/Human-wt/Bel/BE00100/2009/G1P[8], JN258939; RVA/Human-wt/Bel/BE00094/2009/G1P[8], JN258906; Hu/RUS/O884/2010/P[8], JX867351; RVA/Human-wt/BEL/BE00041/2007/G1P[8], HQ392427; RVA/Human-wt/BEL/BE00028/2007/G1P[8], HQ392228; RVA/Human-wt/BEL/BE00027/2008/G1P[8], HQ392218; RVA/Human-wt/BEL/BE00032/2008/G1P[8], HQ392274; RVA/Human-wt/BEL/BE00024/2007/G1P[8], HQ392200; RVA/Human-wt/BEL/BE00014/2006/G1P[8], HQ392083; RVA/Human-wt/BEL/BE00016/2006/G1P[8], HQ392106; RVA/Human-wt/USA/2007719720/2007/G1P[8], JN258335; US0408, EF426119; US9810, EF426127; RVA/Human-wt/Bel/BE00061/2000/G1P[8], KC193627; RUSNov061486, JQ230065; Nov072303, GQ477092; Nov10N447, HQ611018; Hu/RUS/Nov10N552/G3P[8], JQ230093; Hu/RUS/O903/2010/P[8], JX867352; Nov10N93, HQ611003; Hu/RUS/O938/2010/G1P[8], JX867359; Hu/RUS/Nov071971/2007/G1P[8], JQ713098; Hu/RUS/Nov072058/2007/G1P[8], JQ585600; RVA/Human-wt/BEL/BE00025/2007/G1P[8], HQ392205; Nov09B23, GQ477116; Omsk08423, HQ738596; Omsk08454, GU390450; Nov07K6, GQ477094; Nov071767, JQ230066; Hu/RUS/Nov061255/2006/G4P[8], JQ585598; Hu/RUS/Omsk08425/2008/G1P[8], JQ818154; RVA/Human-wt/GR/Ath144/2010/G4P[8], KC890878; RVA/Human-wt/GR/Ath198/2008/G4P[8], KC890876; RVA/Human-wt/GR/Ath193/2009/G4P[8], KC890880; RVA/Human-wt/GR/Ath113/2009/G4P[8], KC890877; RVA/Human-wt/GR/Ath186/2009/G4P[8], KC890879; RVA/Human-wt/GR/Ath146/2010/G4P[8], KC890881; RUSNov083260, GQ477098; Nov082884, GU390440; Nov0526, GU390426; RUSNov05101, GQ468563; Nov04H578, GQ468561; Nov04H510, JQ230061; RUSNov04H672, JQ230062; Nov09D100, GU390453; Nov09D91, GQ477132; Nov09B56, GQ477120; Hu/RUS/Nov11N2903/2011/P[8], JX841143; Nov0576, GU390428; Nov06974, GQ477079; RUSNov04H508, JQ230060; Nov05688, GQ468567; Hu/RUS/Nov04H641/2004/G9, JQ818165; RUSNov05237, JQ230063; Nov05701, GQ468568; Nov072332, HQ445975; Nov061329, JQ248938; Hu/RUS/Nov03H233/2003/G1P[8], JQ818159; Hu/RUS/Nov03H257/2003/G1P[8], JQ818161; RUSNov083427, GQ477103; Nov05177, GU390438; Nov05114, GU390431; Nov04H360, JQ230059; RUSNov04H390, GQ468558; Nov09D57, GQ477128; Nov082887, GU390441; Hu/RUS/Nov10N312/2010/G2G4P[4], JQ613167; Hu/RUS/Nov10N404/2010, JQ585602; Omsk09526, HQ738599; Hu/RUS/Nov10N511/G4P[8], JQ230091; Hu/RUS/Nov09D221/G4P[8], JQ230074; Hu/RUS/Omsk10597/2010/G4P[8], JQ818155; Hu/RUS/O626/2010/G4P[8], JQ951795; Hu/RUS/Nov11N2826/2011/G4P[8], JX841142; Hu/RUS/Nov10N632/2010/G4P[8], JQ818168; Nov10N692, HQ611030;

Hu/RUS/Nov10N1008/G4P[8], JQ230096; Nov10N596, HQ611025; Hu/RUS/Nov10N164/G4P[8], JQ230085; Hu/RUS/Nov09D336/G4P[8], JQ230081; Hu/RUS/Nov10N123/G4P[8], JQ230084; Nov10N331, HQ611010; Nov10N282, HQ611008; Hu/RUS/O672/2010/G4P[8], JX867366; Hu/RUS/Nov10N348/G4P[8], JQ230087; Nov10N383, HQ611013; Omsk10574, HQ738601; Hu/RUS/Nov11N2709/G4P[8], JQ230100; Hu/RUS/O1005/2010/G4P[8], JX867365; Hu/RUS/O646/2010/G4P[8], JX867358; Hu/RUS/Nov11N3220/2011/G3P[8], JX841180; Nov10N565, HQ611033; Nov10N478, HQ611022; Nov09D263, GU390460; Nov10N92, HQ611002; Hu/RUS/O979/2010/G4P[8], JX867364; Hu/RUS/Nov10N344/2010/G2G4P[8], JQ613168; GER12608, FJ747617; Hu/RUS/O895/2011/G1P[8], JX867368; Hu/RUS/O933/2010/G1P[8], JX867356; Hu/RUS/O868/2010/G1P[8], JX867353; Hu/RUS/O638/2010/G1P[8], JX867349; 0613158CA, EU984108; Hu/RUS/O1015/2010/G1P[8], JX867362; Hu/RUS/O1034/2010/G1P[8], JX867363; RVA/Human-wt/AUS/CK00085/2008/G1P[8], JX027842; RVA/Human-wt/AUS/CK00084/2008/G1P[8], JX027830; Nov09D83, GQ477131; RVA/Human-wt/USA/2007719685/2007/G1P[8], JN258348; RVA/Human-wt/BEL/BE00009/2005/G1P[8], HQ392041; RVA/Human-wt/BEL/BE00020/2006/G1P[8], HQ392152; RVA/Human-wt/BEL/BE00012/2006/G1P[8], HQ392059; RVA/Human-wt/Bel/BE00055/1999/G1P[8], JN258792; human/Vanderbilt/VU060729/2006/G1P[8], JF490849; human/Vanderbilt/VU060730/2006/G1P[8], JF490859; RVA/Human-wt/USA2008747100/2008/G1P[8], HM773793; RVA/Human-wt/USA2008747106/2008/G1P[8], HM773782; LB2758, HM467948; human/Victoria/CK00003/2004/G1P[8], JF490112; human/Victoria/CK00004/2004/G1P[8], JF490123; human/Vanderbilt/VU050626/2005/G1P[8], JF490661; human/Vanderbilt/VU050627/2005/G1P[8], JF490673; RVA/Human-wt/USA2007744509/2007/G1P[8], HM773815; RVA/Human-wt/USA2007744510/2007/G1P[8], HM773804; RVA/Human-wt/USA2007719698/2007/G1P[8], HM773771; human/Victoria/CK00034/2007/G1P[8], JF490408; human/Vanderbilt/VU050676/2005/G1P[8], JF490838; RVA/Human-wt/BEL/BE00010/2006/G1P[8], HQ392052; human/Vanderbilt/VU060733/2006/G1P[8], JF490948; RVA/Human-wt/AUS/CK00072/2007/G1P[8], JX027743; RVA/Human-wt/AUS/CK00053/2007/G1P[8], JX027663; RVA/Human-wt/USA/2008747288/2008/G1P[8], JN258382; RVA/Human-wt/AUS/CK00071/2007/G1P[8], KC769430; RVA/Human-wt/AUS/CK00070/2007/G1P[8], KC769419; RVA/Human-wt/AUS/CK00055/2007/G1P[8], KC769309; RVA/Human-wt/BEL/BE00030/2008/G1P[8], HQ392252; RVA/Human-wt/BEL/BE00033/2008/G1P[8], HQ392285; human/Vanderbilt/VU050643/2005/G1P[8], JF490714; RVA/Human-wt/USA/DC5385/1991/G1P[8], KC579673; RVA/Human-wt/USA/DC5406/1991/G1P[8], KC579763; RVA/Human-wt/USA/DC5404/1991/G1P[8], KC579717; RVA/Human-wt/USA/DC5411/1991/G1P[8], KC580472; RVA/Human-wt/USA/DC5405/1991/G1P[8], KC579976; human/Bethesda/DC5751/1991/G3P[8], FJ947798; RVA/Human-wt/USA/DC5387/1991/G1P[8], KC580560; RVA/Human-wt/USA/DC5362/1991/G1P[8], KC442918; RVA/Human-wt/USA/DC5390/1991/G1P[8], KC579847; RVA/Human-wt/USA/DC5423/1991/G1P[8], KC580416; RUSNov03H173, JQ230058; Nov03H136, GU390415; Omsk0798, GU390444; Omsk07102, GU390445; Hu/RUS/Nov061368/2006/G4P[8], JQ713097; Nov04H502, GU390422; RVA/humantc/USADC/DC706/1980/G9P[8], JF521466; RVA/humantc/USADC/G2275/1980/G9P[8], JF521477;

RVA/Humantc/IDN/57M/1980/G4P[10], JQ863313; human/Bethesda/DC1563/1974/G3P[8], FJ947180; RVA/Humantc/USA/P/1974/G3P1A[8], EF583040; RVA/Human/JPN/Hosokawa/1983/G4P1A[8], DQ870492; human/Bethesda/DC4613/1980/G4P[8], HM773914; human/Bethesda/DC1285/1980/G4P[8], FJ947169; RVA/Humantc/GBR/ST3/1975/G4P2A[6]EF583048; RVA/Humantc/USA/WI61/1983/G9P1A[8], EF583052; RV3, FJ998275; HR3RV3, U04741; RVA/Humantc/AUS/McN13/1980/G3P2A[6], JX416221; Hu/RUS/Nov04H297/2004/G1P[8], JQ818163; Hu/RUS/Nov03H269/2003/G1P[8], JQ818162; Hu/RUS/Nov03H223/2003/G1P[8], JQ818158; Hu/RUS/Nov11N2817/G4P[8], JQ230101; CMH110/07, JQ043299; CMH060/07, JQ043297; Omsk08350, GQ477110; Nov10N415, HQ611016; Nov10N375, HQ611014; CU957KK/11, JN706552; CU956KK/11, JN706551; CU875BK/10, JN706550; Hu/RUS/O917/2010/G1P[8], JX867347; human/Victoria/CK00019/2005/G1P[8], JF490265; human/Victoria/CK00020/2005/G1P[8], JF490277; human/Vanderbilt/VU050657/2005/G1P[8], JF490764; human/Victoria/CK00015/2005/G1P[8], JF490222; human/Victoria/CK00016/2005/G1P[8], JF490233; RVA/Human-wt/USA/2008747323/2008/G1P[8], JN258392; RVA/Human-wt/ITA/AV28/2010/G9P8, JX195078; RVA/Human-wt/BEL/BE00045/2009/G1P[8], HQ392404; RVA/Human-wt/BEL/BE00031/2008/G1P[8], HQ392262; RVA/Human-wt/ITA/AV21/2010/G9P8, JX195067; RVA/Human-wt/USA2007719907/2007/G1P[8], HM773848; RVA/Human-wt/USA2007719945/2007/G1P[8], HM773837; human/Vanderbilt/VU060732/2006/G1P[8], JF490937; human/Vanderbilt/VU050640/2005/G1P[8], JF490704; human/Vanderbilt/VU050613/2005/G1P[8], JF490584; human/Vanderbilt/VU050619/2005/G1P[8], JF490629; human/Vanderbilt/VU050620/2005/G1P[8], JF490640; human/Vanderbilt/VU050633/2005/G1P[8], JF490693; human/Vanderbilt/VU050623/2005/G1P[8], JF490650; human/Vanderbilt/VU05062/2005/G1P[8], JF490563; human/Vanderbilt/VU050667/2005/G1P[8], JF490794; US6588, FJ152109; CAU160, EU679384; CAU200, EU679387; RVA/Human-wt/AUS/CK00086/2009/G1P[8], JX027852; Hu/CI81/2011/KOR, JN887819; CAU163, EU679385; CAU136, EU679383; RVA/Human-wt/BEL/BE00004/2005/G1P[8], HQ392007; CAU214, EU556222; CAU195, EU556221; RVA/Human-wt/BEL/B4633/2003/G12P[8], DQ146642; RVA/Human-wt/USA2008747307/2008/G9P[8], HM773639; RVA/Human-wt/BEL/BE00044/2009/G1P[8], HQ392389; RVA/Human-wt/ZMB/MRCDPRU3491/2009/G12P[6], KF636150; RVA/Human-wt/Bel/BE00090/2003/G1P[8], JN258880; RVA/Human-wt/Bel/BE00112/2009/G1P[8], JN258948; human/Bethesda/DC1730/1979/G3P[8], FJ947323; human/Bethesda/DC1359/1980/G4P[8], HM773870; RVA/Human-wt/USA/DC4315/1988/G1P[8], KC579654; RVA/Human-wt/USA/DC3975/1988/G1P[8], KC580258; RVA/Human-wt/USA/DC4089/1988/G1P[8], KC580361; RVA/Human-wt/USA/DC4092/1988/G1P[8], KC579479; RVA/Human-wt/USA/DC4094/1988/G1P[8], KC579524; RVA/Human-wt/USA/DC4065/1988/G1P[8], KC579579; RVA/Human-wt/USA/DC4081/1988/G1P[8], KC579612; RVA/Human-wt/USA/DC3723/1989/G1P[8], KC579706; RVA/Human-wt/USA/DC4087/1988/G1P[8], KC579750; RVA/Human-wt/USA/DC4005/1988/G1P[8], KC579774; RVA/Human-wt/USA/DC3687/1989/G1P[8], KC579818; RVA/Human-wt/USA/DC4085/1988/G1P[8], KC579859; RVA/Human-wt/USA/DC3827/1989/G1P[8], KC579998; RVA/Human-wt/USA/DC4053/1988/G1P[8],

KC580020; RVA/Human-wt/USA/DC4057/1988/G1P[8], KC580090; RVA/Human-wt/USA/JP00299/1989/G1P[8], KC580158; RVA/Human-wt/USA/DC4086/1988/G1P[8], KC580181; RVA/Human-wt/USA/DC4080/1988/G1P[8], KC580236; RVA/Human-wt/USA/DC3985/1988/G1P[8], KC580247; RVA/Human-wt/USA/DC4064/1988/G1P[8], KC580394; RVA/Human-wt/USA/DC4083/1988/G1P[8], KC580405; RVA/Human-wt/USA/DC4066/1988/G1P[8], KC580460; RVA/Human-wt/USA/DC4038/1988/G1P[8], KC580483; RVA/Human-wt/USA/DC3980/1988/G1P[8], KC580507; RVA/Human-wt/USA/DC4084/1988/G1P[8], KC580518; RVA/Human-wt/USA/DC3976/1988/G1P[8], KC580541; RVA/Human-wt/USA/DC3684/1989/G1P[8], KC580582; RVA/Human-wt/USA/DC3956/1988/G1P[8], KC580605; RVA/Human-wt/USA/DC4312/1988/G1P[8], KC579937; RVA/Human-wt/USA/DC3721/1989/G1P[8], KC579796; human/Bethesda/DC5115/1977/G4P[8], HM773947; human/Bethesda/DC5064/1977/G4P[8], HM773936; RVA/Human-wt/USA/DC1108/1977/G1P[8], KC580066; human/Bethesda/DC4996/1977/G4P[8], HM773925; human/Bethesda/DC2241/1977/G4P[8], HM773881; RVA/Human-wt/USA/DC1127/1977/G1P[8], KC580290; human/Bethesda/DC827/1978/G4P[8], HM773958; RUSNov083428, GQ477104; Nov09D10, GQ477123; Omsk08381, GU390449; Nov083149, HQ445978; Nov083296, GU390443; RVA/Human-wt/USA/DC1117/1977/G1P[8], KC580350; RVA/Human-wt/USA/DC1116/1977/G1P[8], KC580571; RVA/Human-wt/USA/DC1141/1977/G1P[8], KC580627; RVA/Human-wt/USA/DC1007/1978/G1P[8], KC579601; RVA/Human-wt/USA/DC1083/1977/G1P[8], KC579785; RVA/Human-wt/USA/DC1108/1977/G1P[8], KC580067; RVA/Human-wt/USA/DC1127/1977/G1P[8], KC580289; RVA/Human-wt/USA/DC1179/1978/G1P[8], KC579590; RVA/Human-wt/USA/DC1164/1978/G1P[8], KC579623; RUSNov072253, GQ477091; Nov10N327, HQ611009; Omsk0779, GQ477107; NIV929893, FJ685614; human/Bethesda/DC2102/1976/G3P[8], FJ947831; human/Bethesda/DC133/1976/G3P[8], FJ947268; human/Bethesda/DC1496/1976/G3P[8], FJ947290; human/Bethesda/DC2171/1976/G3P[8], FJ947411; human/Bethesda/DC2266/1976/G3P[8], FJ947886; human/Bethesda/DC2212/1976/G3P[8], FJ947853; human/Bethesda/DC23/1976/G3P[8], FJ947213; human/Bethesda/DC1505/1976/G3P[8], FJ947356; human/Bethesda/DC2238/1976/G3P[8], FJ947422; human/Bethesda/DC2114/1976/G3P[8], FJ947389; human/Bethesda/DC139/1976/G3P[8], FJ947224; human/Bethesda/DC129/1976/G3P[8], FJ947235; RVA/Humantc/JPN/K8/1977/G1P[9], JQ713648; RVA/Humantc/JPN/YO/1977/G3P1A[8], DQ870500; RVA/Human-wt/Bel/BE00052/1999/G1P[8], JN258780; RVA/Human-wt/Bel/BE00085/2002/G1P[8], JN258872; RVA/Human-wt/Bel/BE00093/2003/G1P[8], JN258897; RVA/Human-wt/Bel/BE00082/2002/G1P[8], JN258858; human/Victoria/CK00045/2006/G1P[8], JF490500; human/Victoria/CK00030/2006/G1P[8], JF490376; human/Victoria/CK00033/2007/G1P[8], JF490398; human/Victoria/CK00032/2006/G1P[8], JF490387; RVA/Human-wt/AUS/CK00078/2007/G1P[8], JX027798; RVA/Human-wt/AUS/CK00067/2007/G1P[8], JX027731; RVA/Human-wt/AUS/CK00075/2007/G1P[8], JX027765; RVA/Human-wt/AUS/CK00051/2007/G1P[8], JX027638; RVA/Human-wt/AUS/CK00054/2007/G1P[8], JX027677; RVA/Human-wt/AUS/CK00063/2007/G1P[8], JX027720; human/Victoria/CK00041/2006/G1P[8], JF490475; RVA/Human-wt/AUS/CK00052/2007/G1P[8], JX027652; RVA/Human-wt/Bel/BE00067/2000/G1P[8], JN258807; RVA/Human-wt/BEL/BE00013/2006/G1P[8], HQ392076; RVA/Human-

wt/BEL/BE00042/2008/G1P[8], HQ392373; RVA/Human-wt/USA/2007769947/2007/G1P[8], JN258403; RVA/Human-wt/BEL/BE00021/2007/G1P[8], HQ392161; Nov09D47, GQ477127; CAU219, EU679388; E210, U36240; 97'B53, AF260931; Syntheticconstruct, AY803761; mani362/07, HM348747; mani253/07, HM348745; CMP101/01, EU372756; CMP100/01, EU372755; Hu/RUS/Nov11N2541/G4, JQ230098; Hu/RUS/Nov11N1485/G4P[6], JQ230097; Nov10N806, HQ611032; GX54, KF041434; GX82, KF447865; EC2184/ECU/G11P[6], GQ149095; RVA/Human-wt/USA/DC2164/1976/G1P[8], KC579535; RVA/Human-wt/USA/DC2181/1976/G1P[8], KC579918; RVA/Human-wt/USA/DC108/1977/G1P[8], KC580192; human/Victoria/CK00007/2004/G1P[8], JF490156; rotavirussubgroup2, X57943; RVA/Human-wt/USA/DC222/1979/G1P[8], KC579684; RVA/Human-wt/USA/DC255/1979/G1P[8], KC579807; RVA/Human-wt/USA/DC104/1974/G1P[8], KC579695; RVA/Humantc/USA/D/1974/G1P1A[8], EF583024; RVA/Human-wt/USA/DC1228/1980/G1P[8], KC579896; RVA/Human-wt/USA/DC1292/1980/G1P[8], KC580438; Wa, K02086; RVA/Human-wt/USA/DC435/1975/G1P[8], KC579728; RVA/Human-wt/USA/DC397/1975/G1P[8], KC579829; RVA/Human-wt/USA/DC2922/1975/G1P[8], KC579884; RVA/Human-wt/USA/DC2914/1975/G1P[8], KC579907; RVA/Human-wt/USA/DC2340/1975/G1P[8], KC580101; RVA/Human-wt/USA/DC1448/1975/G1P[8], KC580203; RVA/Human-wt/USA/DC429/1975/G1P[8], KC580269; RVA/Human-wt/USA/DC436/1974/G1P[8], KC580301; RVA/Human-wt/USA/DC496/1974/G1P[8], KC580372; RVA/Human-wt/USA/DC444/1975/G1P[8], KC580383; RVA/Human-wt/USA/DC419/1975/G1P[8], KC580449; Wag5re, FJ423150; Wag7/8re, FJ423140; TCParWa, FJ423129; VirWa, FJ423118; RVA/Human-wt/USA/DC484/1974/G1P[8], KC579987; RVA/Human-wt/USA/DC425/1975/G1P[8], KC580135; RVA/Human-wt/USA/DC412/1974/G1P[8], KC579546; RVA/Human-wt/USA/DC799/1978/G1P[8], KC579964; RVA/Human-wt/USA/DC4347/1988/G1P[8], KC579490; RVA/Human-wt/USA/DC4315/1988/G1P[8], KC579655; RVA/Human-wt/BEL/BE00048/2009/G1P[8], HQ392416; RVA/Human-TC/USA/Rotarix/2009/G1P[8], JX943613; RVA/Human-wt/USA/DC3779/1989/G1P[8], KC580046; RVA/Human-wt/USA/DC3853/1989/G1P[8], KC580169; RVA/Human-wt/USA/DC3855/1989/G1P[8], KC580328; RVA/Human-wt/USA/DC3828/1989/G1P[8], KC580616; RVA/Human-wt/USA/DC3859/1989/G1P[8], KC580214; RVA/Human-wt/USA/DC3669/1989/G1P[8], KC579513; RVA/Human-wt/USA/DC5413/1991/G1P[8], KC580312; mcs/1307, EU753964; Hu/RUS/Nov11N2687/G4P[6], JQ230099; Nov10N459, HQ611020; RVA/Humantc/BRA/IAL28/1992/G5P[8], EF583032; human/Bethesda/CH5470/1991/G3P[8], FJ947908; human/Bethesda/CH5484/1991/G3P[8], FJ947477; RVA/Human-wt/USA/DC5385/1991/G1P[8], KC579672; RVA/Human-wt/USA/DC5390/1991/G1P[8], KC579848; human/Bethesda/DC5491/1991/G3P[8], FJ947499; human/Bethesda/CH5488/1991/G3P[8], FJ947488; human/Bethesda/CH5477/1991/G3P[8], FJ947466; human/Bethesda/CH5498/1991/G3P[8], FJ947930; human/Bethesda/CH5483/1991/G3P[8], FJ947919; human/Bethesda/CH5455/1991/G3P[8], FJ947897; RVA/Human-wt/USA/DC5387/1991/G1P[8], KC580559; human/Bethesda/DC5549/1991/G3P[8], FJ947521; human/Bethesda/DC5544/1991/G3P[8], FJ947510; human/Bethesda/DC5553/1991/G3P[8], FJ947941; human/Bethesda/DC5710/1991/G3P[8], FJ947787; human/Bethesda/CH5459/1991/G3P[8], FJ947444; human/Bethesda/CH5475/1991/G3P[8], FJ947455; human/Bethesda/CH5446/1991/G3P[8], FJ947433; human/Bethesda/DC5728/1991/G3P[8],

FJ947334; US6253, EF426131; RVA/Human-wt/USA/DC4312/1988/G1P[8], KC579938; RVA/Human-wt/USA/DC4352/1988/G1P[8], KC580078; RVA/Human-wt/USA/DC4360/1988/G1P[8], KC580594; RVA/Human-wt/USA/DC4345/1988/G1P[8], KC580638; human/Bethesda/DC4320/1988/G4P[8], HM773892; RVA/Human-wt/USA/DC1212/1980/G1P[8], KC579739; RVA/Human-wt/USA/DC1260/1980/G1P[8], KC580496; human/Bethesda/DC1600/1980/G3P[8], FJ947776; human/Bethesda/DC792/1980/G3P[8], FJ947765; human/Bethesda/DC1208/1980/G4P[8], HM773859; human/Bethesda/DC4608/1980/G4P[8], HM773903; CJN, AF461757; RVA/Human-wt/USA/DC576/1979/G1P[8], KC580339; RVA/Human-wt/USA/DC527/1979/G1P[8], KC580427; RVA/Human-wt/USA/DC247/1979/G1P[8], KC580225; RVA/Human-wt/USA/DC578/1979/G1P[8], KC579873; RVA/Human-wt/USA/DC582/1979/G1P[8], KC579502; RVA/Human-wt/USA/DC1476/1974/G1P[8], KC579568; RVA/Human-wt/USA/DC581/1979/G1P[8], KC580113; RVA/Human-wt/USA/DC273/1979/G1P[8], KC580530; RVA/Human-wt/USA/DC570/1979/G1P[8], KC579634; RVA/Human-wt/USA/DC2314/1976/G1P[8], KC579557; RVA/Human-wt/USA/DC102/1974/G1P[8], KC580009; RVA/Human-wt/USA/DC4038/1988/G1P[8], KC580484; KU, AB022768; 116E/AG, FJ361206; GAU85998, U85998; RVA/Human-tc/VEN/M37/1982/G1P2A[6], JX416207; RVA/Human-wt/USA/DC1260/1980/G1P[8], KC580495; RVA/Human-wt/USA/DC1230/1980/G1P[8], KC579953; human/Bethesda/DC2109/1976/G3P[8], FJ947378; human/Bethesda/DC1497/1976/G3P[8], FJ947345; human/Bethesda/DC131/1976/G3P[8], FJ947257; human/Bethesda/DC168/1976/G3P[8], FJ947754; human/Bethesda/DC2119/1976/G3P[8], FJ947400; human/Bethesda/DC5142/1975/G3P[8], FJ947202; human/Bethesda/DC2106/1976/G3P[8], FJ947842; human/Bethesda/DC4772/1976/G3P[8], FJ947367; human/Bethesda/DC1898/1976/G3P[8], FJ947301; human/Bethesda/DC2081/1976/G3P[8], FJ947820; human/Bethesda/DC2069/1976/G3P[8], FJ947809; human/Bethesda/DC1494/1976/G3P[8], FJ947279; human/Bethesda/DC1455/1975/G3P[8], FJ947191; RVA/Human-wt/USA/DC1210/1980/G1P[8], KC580124; human/Bethesda/DC2262/1976/G3P[8], FJ947875; human/Bethesda/DC130/1976/G3P[8], FJ947246; human/Bethesda/DC2239/1976/G3P[8], FJ947864; human/Bethesda/DC135/1979/G3P[8], FJ947312; Gottfried, D00326; RVA/Human/NCA/18J/2010/G1P[8], JN129100; RVA/Human/NCA/25J/2010/G1P[8], JN129103; RVA/Human/NCA/22J/2010/G1P[8], JN129101; RVA/Human/NCA/26J/2010/G1P[8], JN129104; RVA/Human/NCA/24J/2010/G1P[8], JN129102; RVA/Human/NCA/OL/2010/G4P[6]JN129111; mani265/07, HM348746; RVA/Horse-tc/GBR/L338/1991/G13P[18]JF712559; L338, D82974; RVA/Horse-tc/JPN/OH-4/1982/G6P[5], KC815673; HO-5, D82973; R-13, D82976; FI14, D00323; R-3, D82978; HI-23, D82972; RVA/Horsewt/IRL/03V04954/2003/G3P[12]JN903520; RVA/Horsewt/ARG/E30/1993/G3P[12]JF712570; H1, AF242394; OSU, AF317123; A131, AF317124; A253, AF317122; YM, X69487; A411, AF317125; KJ251, HM988972; CMP34/00, EU372782; CMP034, DQ534018; CMP39/00, EU372751; K71, JX971583; K5, JX971573; KV0407, EU873010; DN30209, JN977137; JL94, AY538664; CH-1, GU188283; GD, FJ617209; PRG9121, JF796738; RMC/G60, AY601552; RMC321, AF531913; RMC/G7, AY601551; mani97/06, HM348744; RU172, DQ204741; Tma, JF970185; R479, DQ873675; pig/China/NMTL/2008/G9P[23], JF781162; 4S, L29186; 4FL29184; 2010/WH-a, JN034041; LL3354, EU330646; CRW-8, U82971; CN86, U10031; 82B, JN974814; ZZ12, KF303566;

Hu/BEL/BE2001/2009/G9P[6], JQ993320; CMP67/02, EU372770; CMP54/02, EU372766; PoRV-1/CBNU1, JQ343834; PRG9235, JF796705; PRG942, JF796727; PRG921, JF796716; CMP74/01, EU372790; CMP56/01, EU372789; CMP46/01, EU372787; CMP53/01, EU372759; CMP55/01, EU372788; CMP82/01, EU372791; CMP52/01, EU372758; CMP16/02, EU372762; CMP73/01, EU372761; CMP17/02, EU372763; CMP66/01, EU372760; CMP90/01, EU372792; Mc345, JN104622; Mc323, JN104614; CMP45/, 08HQ268858; Ryukyu1120, AB741653; F8P4A, JN974815; F8P4B, JN974816; F7P4, JN974812; ROTA01, KC855060; ROTA02, KC855061; ROTA03, KC855062; RVA/Pig-wt/THA/CMP29/08/2008/G3P[13], AB779620; RVA/Pig-wt/THA/CMP40/08/2008/G3P[23], AB779621; CMP48/08, HQ268859; CMP86/02, EU372796; CMP10/03, EU372781; CMP11/03, EU372797; CMP66/02, EU372794; CMP12/03, EU372798; CMP12/01, EU372783; CMP127/01, EU372757; CMP16/03, EU372799; CMP31/01, EU372786; CMP27/01, EU372784; CMP34/01, EU372754; CMP33/01, EU372753; CMP40/02, EU372765; CMP39/02, EU372764; CMP65/02, EU372769; CMP25/01, EU372752; CMP68/02, EU372771; CMP29/01, EU372785; CMP107/02, EU372775; CMP57/02, EU372767; CMP113/02, EU372777; CMP8/03, EU372780; CMP6/03, EU372779; CMP109/02, EU372776; CMP96/02, EU372773; CMP95/02, EU372772; CMP104/02, EU372774; CMP3/03, EU372778; CMP83/02EU372795; CMP64/02EU372768; CMP105/01EU372793; RVA/Human-wt/KOR/CAU122/2012/G11P[25], KC140589; RVA/Human-wt/NPL/KTM368/2004/G11P[25], GU199496; RVA/Human-wt/IND/N38/2009/G11P[25]JX040423; Human/LL4260/China/I12, KC149926; Po/CEM060003/Canada/2006/G2P[27]I14, GU183245; 82A, JN974813; EMcN, AY267007; EBPo/G16P[16], JF309301; ETD822, GQ479952; MRU65988, U65988; MRU36474, U36474; EO, AY947543; RVA/Bat/MSLH14/G3P[3], KC960623; CMH079/05, EU791923; CMH222DQ288659; N5, JQ423906; HCR3A.EU708905; A7910/G3P[3], EU708938; Ro1845, EU708894; K9, EU708927; Cat97/G3P[3], EU708949; CU1, EU708916; 6212, JF804993; RVA/Horsewt/ARG/E3198/2008/G3P[3], JX036369; Cat2/G3P[9], EU708960; RVA/Humantc/JPN/AU1/1982/G3P3[9,]DQ490538; CMH120/04, DQ923796; CMH134/04, DQ923800; RVA/Humantc/CHN/L621/2006/G3P[9], JX946162; CU365KK/08, JN706533; RVA/Human-wt/CHN/E2451/2011/G3P[9], JX946172; RVA/Humantc/ITA/PA26097/1997/G3P[3], HQ661116; RVA/Dogtc/ITA/RV19895/1995/G3P[3]HQ661138; RVA/Dogtc/ITA/RV5296/1996/G3P[3], HQ661127; RVA/Humantc/THA/T152/1998/G12P[9], DQ146702; B10, HM627557; RVA/Rhesustc/USA/TUCH/2002/G3P[24], AY594670; bat/4852/Kenya/2007, GU983675; RVA/Horsewt/ARG/E403/2006/G14P[12], JF712581; RVA/Horsewt/ARG/E4040/2008/G14P[12], JN872869; RVA/Horse-tc/JPN/OH-4/1982/G6P[5], KC815684; RVA/Horse-tc/JPN/CH-3/1987/G14P[12]KC815697; H-2, D00324; RVA/Horsewt/ZAF/EqRVSA1/2006/G14P[12], JQ345494; RVA/Horsewt/IRL/04V2024/2004/G14P[12], JN903519; FI-23, D82971; RVA/Cow-wt/ARG/B383/1998/G15P[11], FJ347115; S2, Y00437; RVA/Humantc/JPN/S2/1980/G2P1B[4], DQ870488; TBChen, AY787645; CMH171/01, EU372725; DS-1, EF583028; RVA/Human-tc/USA/DS1/1976/G2P1B[4], DQ870507; RVA/Human-wt/AUS/CK20001/1977/G2P[4], KC443591; RVA/Human-tc/PHL/L26/1987/G12P[4], EF583036; RVA/Human-tc/PHL/L26/1987/G12P[4], DQ146695; AK26, JF304930; PAI11/1996, KC178799; XL, JQ004974; Lamb_NT, FJ031028; CC08121/2008, HQ834200; Lp14, L11595; RUBV117, EF200569; RUBV51, EF200567; RVA/Simian-tc/ZAF/SA11N2/1958/G3P[2], JN827249; RVA/Simian-tc/ZAF/SA11N5/1958/G3P[2], JQ688678; RVA/Simian-tc/ZAF/SA11H96/1958/G3P5B[2], DQ838650; Simianrotavirus, X00421;

SA1130/19(SA11g4Oagent[30/19]), DQ838648; SA115N(SA11g4Oagent[5N]), DQ838646; ZTR5, JF896469; SA1130/1A(SA11g4Oagentg5delC[30/1A]), DQ838649; SA115S(SA11g4Oagentg5delC[5S]), DQ838647; A/SA11, AY187029; SA11(H96), JF791806; SA11Ramiglab, L33365; (SA11), M27824; SA11temperature-sensitivemutantG, L15384; AzuK1, AB573082; Dai10, AB573073; 1076, D00325; D205, JF304919; RUSNov06K10, GQ477087; Hu/RUS/O211/2007/G3P[9], JX867343; RUSNov06K2, GQ477086; KF17, JF421979; Hu/RUS/Nov10N507/G3P[9], JQ230092; Hu/RUS/O1154/2011/G3P[9]JX867344; RVA/Human-wt/ITA/PAH136/19, 96/G3P[9]GU296428; RVA/Human-wt/HUN/Hun5/199, 7/G6P[14]EF554108; RVA/Cat-wt/ITA/BA222/2005/G3P[9], GU827410; RVA/Human-wt/TUN/17237/2008/G6P[9], JX271005; Omsk08442, JQ230102; RVA/Human-wt/BEL/B4106/2000/G3, P[14]AY740737; RVA/Rabbit-tc/ITA/3096/1996/G3P[14], DQ205226; RVA/Cow-wt/ZAF/1603/2007/G6P[5], JN831213; RVA/Cow-wt/ZAF/1605/2007/G6P[5], JN831235; RVA/Rabbit-tc/NLD/K1130027/2011/G6P[11], KC488889; 22R, AB040055; RVA/Human-tc/IND/69M/1980/G8P4[10], EF583016; bovine-tc/SouthAfrica/'O'Agent/1965/G8P[1], JF693042; RVA/Human-tc/AUS/MG6/1993/G6P[14], EF554097; RVA/Human-wt/COD/DRC88/2003/G8P[8], DQ005110; RVA/Human-wt/COD/DRC86/2003/G8P[6], DQ005121; RVA/Human-wt/SWZ/MRCDPRU4390/2010/G8G9P[6], KF636349; RVA/Human-wt/ZMB/MRCDPRU3463/2009/G8P[4], KF636315; RVA/Human-wt/ZMB/MRCDPRU1621/2008/G8P[4], KF636361; RVA/Human-wt/KEN/MRCDPRU1606/2009/G8P[4], KF636249; Hu/MAL81/G8P[4], JN591410; IS2, X94617; CMH008/05, GU288636; RVA/Human-wt/AUS/336190/2004/G2P4, KC834700; US1205, AF079357; 10924/99, FJ183358; US6259, EF426123; RVA/Human-wt/AUS/CK20022/2001/G2P[4], KC443778; US8720, EF426124; US8922EF426132; Nov04H429, GQ468559; US8635EF426133; MRC-DPRU9317, JN605441; 11531_05AC, HM066129; 11830_06AC, HM123818; 11837_06AC, HM066130; RVA/Human-wt/BGD/N26/2002/G12P[6], DQ146686; RVA/Human-wt/AUS/CK20038/2008/G6P[4], KC443371; GER1H09, GQ414544; RVA/Human-wt/AUS/CK20052/2010/G2P[4], KC443624; RVA/Human-wt/AUS/CK20049/2010/G2P[4], KC443558; human/AUS/RCH041/2010/G2P4, JX965143; RVA/Human-wt/AUS/CK20040/2010/G2P[4], KC443215; RVA/Human-wt/USA/2007769964/2007/G2P[4,]KC442907; RVA/Human-wt/AUS/CK20060/2010/G2P[4]KC443382; PA84/2008, KC178805; PA3/2004, KC178800; RVA/Human-wt/ZAF/MRCDPRU1061/2009/G2P[4], KF636326; RVA/Human-wt/AUS/CK20041/2010/G2P[4], KC443690; RVA/Human-wt/AUS/CK20047/2011/G2P[4], KC443360; RVA/Human-wt/AUS/V203/2009/G2P4KC834699; RVA/Human-wt/AUS/CK20034/2006/G2P[4]KC443547; CMH190/01, EU372726; RVA/Human-wt/AUS/CK20009/2000/G2P[4]KC443437; RVA/Human-wt/AUS/CK20011/2000/G2P[4]KC443426; RVA/Human-wt/AUS/CK20008/2000/G2P[4]KC443712; RVA/Human-wt/AUS/CK20013/2000/G2P[4]KC443734; RVA/Human-wt/AUS/CK20015/2000/G2P[4], KC443182; RVA/Human-wt/AUS/V233/1999/G2P4, KC834701; 1303906RS, HM066135; 1439707MA, HM123836; 1258506ES, HM123824; 1252206BA, HM123823; 1158105AC, HM123816; 1432207MG, HM123834; 1238906RJ, HM123822; 1178205RJ, HM123817; 1577108PE, HM066142; RVA/Human-wt/USA/VU060736/2006/G2P[4], KC443033; RVA/Human-wt/USA/2007719874/2007/G2P[4], KC442874; LB2744, HM467947; CU438KK/09, JN706537; CU497BK/09, JN706539; CU473BK/09JN706538; CU436KK/09,

JN706536; RVA/Human-wt/AUS/CK20036/2007/G2P[4], KC443525; PA150/2006, KC178801; Nov04H391, GQ468557; 1228706BA, HM066131; 1230106BA, HM123820; RVA/Human-wt/USA/VU050666/2005/G2P[4], KC442989; LB2772, HM467951; 1581108SE, HM066145; 1584008RS, HM066146; 1595308RS, HM123840; 1598308BA, HM066152; 15990_08BA, HM123841; 1538508BA, HM066154; 1583608RS, HM123838; Hu/BEL/F01498/2009/G3P[6], JF460838; Hu/BEL/F01322/2009/G3P[6], JF460827; Hu/USA/06242/2006/G2P[6], JF460816; RVA/Human-wt/BEL/B1711/2002/G6P[6], EF554086; TK119, AY456527; RVA/Human-wt/BGD/RV161/2000/G12P[6], DQ490549; Calcutta, AF309652; RVA/Human-wt/BGD/RV176/2000/G12P[6], DQ490555; TK126, AY456528; RVA/Human-wt/AUS/CK20020/2001/G2P[4], KC443349; RVA/Human-wt/AUS/CK20016/2000/G2P[4], KC443657; RVA/Human-wt/MWI/1473/2001/G8P[4], HQ657142; US8908, EF426140; US5139, EF426130; RVA/Human-wt/AUS/CK20003/2000/G2P[4], KC443745; RVA/Human-wt/AUS/CK20007/2000/G2P[4], KC443723; RUSNov0529, GU390427; Nov0510, GU390425; RUSNov05202, GQ468564; Nov04H431, GQ468560; Nov04H676GQ468562; RVA/Human-wt/USA/VU050645/2005/G2P[4], KC443000; CMH134/05, GU288641; PA17/2008, KC178804; CMH054/05, GU288640; CU209KK/08, JN706535; Hu/MAL88/G12[P6], JN591412; Omsk08336, GU390446; Omsk08475, GQ477113; Omsk08375, HQ738588; Omsk08418, HQ738594; Omsk0787, GU138214; Omsk0788, GQ477108; Nov05137, GU390434; RUSNov06128, 3JQ230064; RVA/Human-wt/AUS/CK20024/2006/G2P[4], KC443701; RVA/Human-wt/AUS/CK20025/2006/G2P[4], KC443668; RVA/Human-wt/AUS/CK20029/2006/G2P[4], KC443569; RVA/Human-wt/AUS/CK20035/2006/G2P[4], KC443404; PA83/2007, KC178802; RVA/Human-wt/ZAF/3203WC/2009/G2P[4], HQ657175; CU110BK/08, JN706534; Human-wt/USA/LB1562/2010/G9P4, KC782523; RVA/Human-wt/AUS/CK20059/2010/G2P[4], KC443756; RVA/Human-wt/AUS/CK20053/2010/G2P[4], KC443259; RVA/Human-wt/AUS/CK20044/2010/G2P[4], KC443160; RVA/Human-wt/AUS/CK20046/2010/G2P[4], KC443646; Human-wt/AUS/SA066/2010/G2P4KC571496; RVA/Human-wt/AUS/CK20045/2010/G2P[4], KC443237; RVA/Human-wt/USA/VU080938/2008/G2P[4], KC442978; RVA/Human-wt/USA/2008747095/2008/G2P[4], KC442896; RVA/Human-wt/AUS/CK20028/2006/G2P[4], KC443635; RVA/Human-wt/AUS/CK20033/2006/G2P[4], KC443338; RVA/Human-wt/AUS/CK20027/2006/G2P[4], KC443767; PA130/2010, KC178807; RVA/Human-wt/IND/mcs65/2011/G8P[4], JX307600; human/AUS/WAPC703/2010/G2P4, JX965142; RVA/Human-wt/AUS/CK20051/2010/G2P[4], KC443788; RVA/Human-wt/IND/mcs63/2011/G8P[4], JX307599; human/AUS/MON008/2010/G2P4, JX965144; RVA/Human-wt/IND/mcs72/2011/G8P[4], JX307601; RVA/Human-wt/IND/mcs60/2011/G3P[10], JQ358766; RVA/Human-wt/USA/VU080911/2008/G2P[4], KC443022; RVA/Human-wt/USA/VU08098/2008/G2P[4]P[8], KC442956; RVA/Human-wt/USA/VU080935/2008/G2P[4], KC442944; RVA/Human-wt/USA/VU080913/2008/G2P[4], KC442967; CMH030/07, JQ043294; MMC88, HQ641367; CMH070/07, JQ043298; CMH028/07, JQ043293; CMH049/07, JQ043296; SK424, EU839963; PA108/2007, KC178803; MMC6, HQ641358; RVA/Human-wt/USA/VU050654/2005/G2P[4], KC443011; LB2764, HM467949; 1284006ES, HM066134; ISO97, EF472949; Nov09D1, GQ477121; Hu/RUS/Omsk08257/2008/G2P[4], JQ818152; Omsk08412, HQ738590; Nov09B39, GQ477119; Nov09KZ, GQ477133; Omsk08464, HQ738591; 1578208MG, HM066143; 1586008RJ, HM066148; 1585908MG, HM066147; 1589808MG, HM066149; PA133/2011, KC178806; 1590008MG, HM066150; 1609909ES, HM066155; Nov10N190, HQ611005;

Hu/RUS/Omsk08380/2008/G2P[4], JQ818153; Omsk08471, HQ738585; RUSNov083277, GQ477100; RVA/Human-wt/USA/2007719869/2007/G2P[4], KC442885; Nov09D2, GQ477122; RVA/Human-wt/IND/mcs90/2011/G8P[4], JX307602; 1586308MA, HM066156; 1606409MA, HM066157; 1434407RS, HM123835; 1426307RS, HM123833; 1366807AL, HM066137; 1366307AL, HM123829; 1589408ES, HM123839; 1389107RJ, HM123832; 1379307SE, HM123831; 1378007RJ, HM123830; 1315106RJ, HM123828; 1234306RJ, HM123821; 1222006RJ, HM123819; 1378807SE, HM066138; 1258906RJ, HM123825; 1264706RJ, HM123826; 1315806ES, HM066136; 1277406RJ, HM123827; 1598808BA, HM066153; 1531108RJ, HM066141; 1430307MG, HM066139; RVA/Human-wt/JPN/OH3625/2012/G1P[8], AB796454; RVA/Human-wt/JPN/OH3493/2012/G1P[8], AB796450; RVA/Human-wt/JPN/OH3385/2012/G1P[8], AB796449; RVA/Human-wt/JPN/OH3506/2012/G1P[8], AB796451; RVA/Human-wt/AUS/CK20055/2010/G2P[4], KC443204; RVA/Human-wt/AUS/CK20048/2011/G2P[4], KC443459; Ind/MP/B48, JF720873; RUBV051, EF200568; RUBV3, EF200565; GO34, GU937881; RVA/Human-wt/IND/N121/2003/G10P[11], KC174914; RVA/Human-wt/IND/N240/2004/G10P[11], KC175010; RVA/Human-wt/IND/N138/2003/G10P[11], KC174925; RVA/Human-wt/IND/N273/2004/G10P[11], KC175021; RVA/Human-wt/IND/N313/2004/G10P[11], KC175284; RVA/Human-wt/IND/N83/2003/G10P[11], KC175098; RVA/Human-wt/IND/N36/2003/G10P[11], KC174870; RVA/Human-wt/IND/N37/2003/G10P[11], KC175087; RVA/Human-wt/IND/N39/2003/G10P[11], KC174881; RVA/Human-wt/IND/N62/2003/G10P[11], KC174892; RVA/Human-wt/IND/N74/2003/G10P[11], KC174903; RVA/Human-wt/IND/N190/2004/G10P[11], KC174936; RVA/Human-wt/IND/N191/2004/G10P[11], KC174947; RVA/Human-wt/IND/N192/2004/G10P[11], KC174958; RVA/Human-wt/IND/N198/2004/G10P[11], KC175185; RVA/Human-wt/IND/N197/2004/G10P[11], KC175174; RVA/Human-wt/IND/N214/2004/G10P[11], KC175207; RVA/Human-wt/IND/N212/2004/G10P[11], KC175196; RVA/Human-wt/IND/N330/2004/G10P[11], KC175065; RVA/Human-wt/IND/N291/2004/G10P[11], KC175043; RVA/Human-wt/IND/N329/2004/G10P[11], KC175054; RVA/Human-wt/IND/N375/2004/G10P[11], KC175076; RVA/Human-wt/IND/N160/2003/G10P[11], KC175119; RVA/Human-wt/IND/N187/2004/G10P[11], KC175141; RVA/Human-wt/IND/N188/2004/G10P[11], KC175152; RVA/Human-wt/IND/N243/2004/G10P[11], KC175240; RVA/Human-wt/IND/N196/2004/G10P[11], KC175163; RVA/Human-wt/IND/N228/2004/G10P[11], KC175218; RVA/Human-wt/IND/N247/2004/G10P[11], KC175251; RVA/Human-wt/IND/N259/2004/G10P[11], KC175262; RVA/Human-wt/IND/N223/2004/G10P[11], KC174999; RVA/Human-wt/IND/N137/2003/G10P[11], KC175108; RVA/Human-wt/IND/N292/2004/G10P[11], KC175273; RVA/Human-wt/IND/N215/2004/G10P[11], KC174989; I321, X94618; RVA/Guanacowt/ARG/Chubut/1999/G8P[14], FJ347104; RVA/Guanacowt/ARG/RioNegro/1998/G8P[1], FJ347126; Ind/UP/Bov1, JF742649; Ind/HR/CC156, JF720879; RVA/Human-wt/IND/N1/2009/G6P[14], JX040422; 86, GU984759; 68, GU984757; 79, GU984758; Ind/HR/BRV133, JF720875; cow/B72/IND/2008/I2, HQ171913; cow/Bov2/UP/India/20086, HQ440218; Bov/Ind/UKD/09/M1, HM235508; cow/B100/IND/2008/I2, HQ171912; HP140, DQ003295; HP113, DQ003294; cow/970/IND/2009/I2, HQ171911; Ind/UKD/P14, JF742650; RVA/Humantc/GBR/A64/1987/G10P11[14], EF583020; RVA/Human-wt/HUN/BP1879/2003/G6P[14], FN665682; RVA/cow/ZAF/MRCDPRU1604/2007/G6P[1],

KF636260; RVA/Cowwt/ZAF/1604/2007/G8P[1], JN831224;
RVA/Antelopewt/ZAF/RC1808/G6P[14], FJ495131; RVA/Human-
wt/BEL/B10925/1997/G6P[14], EF554119; RVA/Human-wt/ZAF/2371WC/2008/G9P[8],,
JN014002; RVA/Sheeptc/ESP/OVR762/2002/G8P[14], EF554152;
RVA/Rhesustc/USA/PTRV/1990/G8P[1], FJ422136; RVA/Human-
wt/HUN/BP1062/2004/G8P[14], FN665693; RVA/Human-wt/AUS/V585/2011/G10P[14],
JX567763; RotashieldDS1xRRV, HQ846870;
RVA/Simiantc/USA/RRV/1975/G3P[3]middlelayer, EU636929; SimianRRV, EF583009;
RotashieldRRV, HQ846848; RotashieldST3xRRV, HQ846881; RotashieldDxRRV, HQ846859;
RVA/Humantc/USA/Se584/1998/G6P[9], EF583044; RVA/Horse-tc/JPN/OH-4/1982/G6P[5],
KC815662; OH4, D82975; Nov10N539, HQ611024; Nov10N413, HQ611017; Nov10N397,
HQ611011; RVA/Humantc/ITA/PA169/1988/G6P[14], EF554130; bovine-
tc/USA/NCDV/1971/G6P[1], JF693031; ROBMCP, K02254;
RVA/Cowtc/USA/NCDV/1967/G6P6[1], DQ870496; porcinerotavirusA, DQ119822; DQ75,
GU384194; B12, HM627546; KV0426, EU873012; KV0418, EU873011R-22, D82977;
PxUKreassortant(UKg9P)BRV3G3, GQ496240; PxUKreassortant(UKg9P), GQ225791;
DxUKreassortant(UKg9D)ROTBRV1, GQ496209; DxUKreassortant(UKg9D)BRV1G1,
GQ496198; DxUKreassortant(UKg9D), GQ225789;
RVA/Vaccine/USA/BRVKC1xUK/2009/G10P[5], KC215544;
RVA/Vaccine/USA/MVSBRV1290xUK/2005/G8P[5], KC215512;
RVA/Vaccine/USA/MVSBRV4/1998/G4P[5], KC215501;
AU32xUKreassortant(UKg9AU32)BRV10G9, GQ496291;
1290xUKreassortant(UKg91290)BRV1290G8, GQ496274;
ST3xUKreassortant(UKg9ST3)BRV4G4, GQ496257; DS1xUKreassortant(UKg9DS1)BRV5G2,
GQ496223; AU32xUKreassortant(UKg9AU32), GQ225794; 1290xUKreassortant(UKg91290),
GQ225793; ST3xUKreassortant(UKg9ST3), GQ225792; DS1xUKreassortant(UKg9DS1),
GQ225790; UKtissuecultureadapted(UKtc, X53667; KJ91, HM988974; KJ192, HM988973;
RVA/Vaccine/USA/RotaTeqSC29/1992/G2P7[5], GU565067;
RVA/Vaccine/USA/RotaTeqWI799/1992/G1P7[5], GU565056;
RVA/Vaccine/USA/RotaTeqWI788/1992/G3P7[5], GU565078; WC3, AF411322; RVA/Human-
wt/AUS/CK20039/2008/G1P[8],, KC443602;
RVA/Vaccine/USA/RotaTeqWI794/1992/G6P1A[8],, GU565045; RVA/Human-
wt/ITA/1110527/2005/G6P[14], EF554141; Sun9, AB374146;
RVA/Camelwt/SDN/MRCDPRU447/2002/G8P[11], KC257095; NCDV, AF317127; HQ09,
JN790188; RVA/Human-wt/ITA/PAI58/1996/G3P[9], GU296429; B223, AF317128; BRV033,
AF317126; RVA/Human-wt/ZAF/2371WC/2008/G9P[8], JN014003; Ecu534, EU805774;
Ch03V0158G3, EU486966; Ch03V0358F3, EU486967; Ch06V0661, EU486969; 02V0002G3,
FJ169858; Ch04V0027G6, EU486968; 02V0002G3, DQ096805; AvRV2, JQ085406; Ch-1,
X98870; RK3, D38099; 993/83, L13765; pheasant-tc/GER/10V0112H5/2010/G23P[37],
JX204815; Ty1, X98871; Ty1, D82980; Tu03V0001E10, EU486964;
turkeytc/GER/03V0002E10/2003/G22P[35], JX204826; Tu03V0002E10, EU486965; TY-3,
D82981; genomiccloneTY3, X98872; Ch2, EU486970; AvianCH2, EF687020; ARO, D16329;
RotavirusC, M88768; Toyama, AB738416; Y122, AB740143; Y121, AB740140; Y084,
AB533512; Y083, AB533511; Y082, AB533510; Y081, AB533509; Y091, AB533513; Icheon,
GU199224; V508, AY795898; RVC/Pigwt/USA/RV0143/2011, KC164677;
RVC/Pigwt/USA/RV0104/2011, KC164674; WD534tc, AF162434; 06-144-2, FJ494691;

chicken-wt/DEU/06V0064/2006, JN034680; chicken-wt/BGD/BS7/2010, JN034683; chicken-wt/BGD/MJ5/2010, JN034685; chicken-wt/BGD/HS58/2010, JN034684; chicken-wt/GBR/06V0274/2006, JN034681; chicken-wt/DEU/06V0047/2006, JN034679; chicken/03V0568/DEU/2003, NC021635; chicken/03V0567/DEU/2003, HQ403604; RUBV282, GQ358715; DB176, GQ358713; RUBV226, GQ358714; Nemuro, AB106542; NIV-005626, JQ904201; Bang544, FJ851392; 10913, JQ904209; NIV-005623, JQ904200; NIV-0948756, JQ904207; NIV-04623, JQ904202; IDH-084, GU377228; 9222, JQ904208; 11037, JQ904210; NIV-957971; 1995; JQ904199; NIV-04624, JQ904203; NIV-0632252; 2006; JQ904204; NIV-1048101, JQ904211; NIV-005625, JN009779; NIV-094456, JN009777; NIV-04622, JN009778; Bang373, AY238389; Bang117, GU391305; WH-1, AY539858; NIV-076222, JQ904205; CAL-1, AB037931; MMR-B1, FJ811827; IC-008, GU377217; NIV-0924341, JQ904206; ADRV, M55982; IDIR, M84456; 9222, JQ904221.

References

1. Marthaler D, Rossow K, Gramer M, Collins J, Goyal S, Tsunemitsu H, et al. Detection of substantial porcine group B rotavirus genetic diversity in the United States, resulting in a modified classification proposal for G genotypes. Virology. 2012;433:85–96. PubMed http://dx.doi.org/10.1016/j.virol.2012.07.006

2. Marthaler D, Rossow K, Culhane M, Collins J, Goyal S, Ciarlet M, et al. Identification, phylogenetic analysis and classification of porcine group C rotavirus VP7 sequences from the United States and Canada. Virology. 2013;446:189–98. PubMed http://dx.doi.org/10.1016/j.virol.2013.08.001

3. Wakuda M, Ide T, Sasaki J, Komoto S, Ishii J, Sanekata T, et al. Porcine rotavirus closely related to novel group of human rotaviruses. Emerg Infect Dis. 2011;17:1491–3. PubMed

4. Alam MM, Kobayashi N, Ishino M, Ahmed MS, Ahmed MU, Paul SK, et al. Genetic analysis of an ADRV-N-like novel rotavirus strain B219 detected in a sporadic case of adult diarrhea in Bangladesh. Arch Virol. 2007;152:199–208. PubMed http://dx.doi.org/10.1007/s00705-006-0831-y

5. Yang H, Makeyev EV, Kang Z, Ji S, Bamford DH, van Dijk AA. Cloning and sequence analysis of dsRNA segments 5, 6 and 7 of a novel non-group A, B, C adult rotavirus that caused an outbreak of gastroenteritis in China. Virus Res. 2004;106:15–26. PubMed http://dx.doi.org/10.1016/j.virusres.2004.05.011

6. Jiang S, Ji S, Tang Q, Cui X, Yang H, Kan B, et al. Molecular characterization of a novel adult diarrhoea rotavirus strain J19 isolated in China and its significance for the evolution and origin of group B rotaviruses. J Gen Virol. 2008;89:2622–9. PubMed http://dx.doi.org/10.1099/vir.0.2008/001933-0

7. Nagashima S, Kobayashi N, Ishino M, Alam MM, Ahmed MU, Paul SK, et al. Whole genomic characterization of a human rotavirus strain B219 belonging to a novel group of the genus Rotavirus. J Med Virol. 2008;80:2023–33. PubMed http://dx.doi.org/10.1002/jmv.21286

8. Thompson JD, Higgins DG, Gibson TJ. CLUSTAL W: improving the sensitivity of progressive multiple sequence alignment through sequence weighting, position-specific gap penalties and weight matrix choice. Nucleic Acids Res. 1994;22:4673–80. PubMed http://dx.doi.org/10.1093/nar/22.22.4673

9. Saitou N, Nei M. The neighbor-joining method: a new method for reconstructing phylogenetic trees. Mol Biol Evol. 1987;4:406–25. PubMed

10. Drummond AJ, Rambaut A. BEAST: Bayesian evolutionary analysis by sampling trees. BMC Evol Biol. 2007;7:214. PubMed http://dx.doi.org/10.1186/1471-2148-7-214

11. Drummond AJ, Ho SY, Phillips MJ, Rambaut A. Relaxed phylogenetics and dating with confidence. PLoS Biol. 2006;4:e88. PubMed http://dx.doi.org/10.1371/journal.pbio.0040088

12. Drummond, AJ, Suchard, MA. 2010. Bayesian random local clocks, or one rate to rule them all. BMC Biol. 8:114–7007–8-114.

13. Drummond AJ, Rambaut A, Shapiro B, Pybus OG. Bayesian coalescent inference of past population dynamics from molecular sequences. Mol Biol Evol. 2005;22:1185–92. PubMed http://dx.doi.org/10.1093/molbev/msi103

14. Drummond AJ, Nicholls GK, Rodrigo AG, Solomon W. Estimating mutation parameters, population history and genealogy simultaneously from temporally spaced sequence data. Genetics. 2002;161:1307–20. PubMed

15. Drummond AJ, Suchard MA, Xie D, Rambaut A. Bayesian phylogenetics with BEAUti and the BEAST 1.7. Mol Biol Evol. 2012;29:1969–73. PubMed http://dx.doi.org/10.1093/molbev/mss075

16. Minin VN, Bloomquist EW, Suchard MA. Smooth skyride through a rough skyline: Bayesian coalescent-based inference of population dynamics. Mol Biol Evol. 2008;25:1459–71. PubMed http://dx.doi.org/10.1093/molbev/msn090

Technical Appendix Table. Distribution of RHV-positive samples

Strain name	Collection date	State	Age, d	RVA result	RVB result	RVC result	GenBank accession no.
RVH/Pig-wt/USA/NC7.64–2/2008/GXP[X]	3/27/2008	North Carolina	7	+	+	+	KF757279
RVH/Pig-wt/USA/MN29.9–2/2006/GXP[X]	11/7/2006	Minnesota	14	+	–	–	KF757260
RVH/Pig-wt/USA/NC7.64–3/2008/GXP[X]	3/27/2008	North Carolina	15	+	+	+	KF757280
RVH/Pig-wt/USA/IA5.39–4/2008/GXP[X]	3/3/2008	Iowa	20	+	+	–	KF757271
RVH/Pig-wt/USA/NC5.61–7/2008/GXP[X]	3/5/2008	North Carolina	20	+	+	+	KF757272
RVH/Pig-wt/USA/IL4.46/2008/GXP[X]	2/20/2008	Illinois	21	+	+	+	KF757268
RVH/Pig-wt/USA/MN32.15/2007/GXP[X]	11/28/2007	Minnesota	28	+	+	–	KF757264
RVH/Pig-wt/USA/MN8.63/2008/GXP[X]	4/7/2008	Minnesota	28	+	+	+	KF757283
RVH/Pig-wt/USA/KS9.5–3/2008/GXP[X]	4/15/2008	Kansas	35	+	+	–	KF757285
RVH/Pig-wt/USA/OK5.68–9/2008/GXP[X]	3/6/2008	Oklahoma	35	+	+	+	KF757274
RVH/Pig-wt/USA/AR7.10–1/2012/GXP[X]	4/6/2012	Arkansas	42	+	+	+	KF757289
RVH/Pig-wt/USA/OK5.68–10/2008/GXP[X]	3/6/2008	Oklahoma	42	+	+	+	KF757275
RVH/Pig-wt/USA/OK7.93–3/2008/GXP[X]	4/1/2008	Oklahoma	42	+	+	+	KF757281
RVH/Pig-wt/USA/TX6.85–3/2008/GXP[X]	3/19/2008	Texas	42	+	+	+	KF757278
RVH/Pig-wt/USA/MO33.5–1/2006/GXP[X]	12/13/2006	Missouri	49	–	+	–	KF757262
RVH/Pig-wt/USA/MN32.79–5/2007/GXP[X]	12/5/2007	Minnesota	21–28	+	+	+	KF757265
RVH/Pig-wt/USA/IA5.5–3/2009/GXP[X]	3/5/2009	Iowa	21–39	+	+	+	KF757287
RVH/Pig-wt/USA/KS6.15–8/2008/GXP[X]	3/12/2008	Kansas	21–42	+	+	+	KF757277
RVH/Pig-wt/USA/NC5.71–3/2008/GXP[X]	3/6/2008	North Carolina	21–42	+	+	–	KF757276
RVH/Pig-wt/USA/MN4.87–5/2008/GXP[X]	2/26/2008	Minnesota	21–55	+	+	+	KF757270
RVH/Pig-wt/USA/NC9.38/2008/GXP[X]	4/14/2008	North Carolina	21–55	+	+	+	KF757284
RVH/Pig-wt/USA/IA5.65–5/2008/GXP[X]	3/5/2008	Iowa	22–42	+	+	+	KF757273
RVH/Pig-wt/USA/MN4.38–3/2008/GXP[X]	2/19/2008	Minnesota	35–49	+	+	+	KF757267
RVH/Pig-wt/USA/MN4.53–6/2008/GXP[X]	2/21/2008	Minnesota	35–55	+	+	+	KF757269
RVH/Pig-wt/USA/FL29.88–2/2006/GXP[X]	11/7/2006	Florida	42–55	+	+	–	KF757261
RVH/Pig-wt/USA/IA33.11/2007/GXP[X]	12/10/2007	Iowa	60	+	+	+	KF757266
RVH/Pig-wt/USA/MN9.65–4/2008/GXP[X]	4/16/2008	Minnesota	63	+	+	+	KF757286
RVH/Pig-wt/USA/AR7.32–4/2009/GXP[X]	3/26/2009	Arkansas	70	+	+	+	KF757288
RVH/Pig-wt/USA/OK8.1–8/2008/GXP[X]	4/3/2008	Oklahoma	84	–	+	+	KF757282
RVH/Pig-wt/USA/MN1.82–12/2007/GXP[X]	1/22/2007	Minnesota	56–112	+	–	+	KF757263

*RHV, rotavirus H; +, positive; –, negative.
